# Stress adaptation under *in vitro* evolution influences survival and metabolic phenotypes of clinical and environmental strains of *Vibrio cholerae* El-Tor

**DOI:** 10.1128/spectrum.01211-24

**Published:** 2025-02-11

**Authors:** Nana Eghele Adade, Stephen Dela Ahator, Inmaculada García-Romero, Macarena Algarañás, Vincent Appiah, Miguel A. Valvano, Samuel Duodu

**Affiliations:** 1 West African Centre for Cell Biology of Infectious Pathogens, College of Basic and Applied Sciences, University of Ghana, Accra, Ghana; 2 Department of Biochemistry, Cell, and Molecular Biology, College of Basic and Applied Sciences, University of Ghana, Accra, Ghana; 3 Infection Biology Group, Wellcome-Wolfson Institute for Experimental Medicine, Queen’s University Belfast, Belfast, United Kingdom; 4 Department of Microbiology, Korle-Bu Teaching Hospital, Accra, Ghana; 5 Centre for New Antibacterial Strategies (CANS) and Research Group for Host-Microbe Interactions, Department of Medical Biology, Faculty of Health Sciences, UiT- The Arctic University of Norway, Tromsø, Norway; 6 Centro Andaluz de Biología del Desarrollo, CSIC-Universidad Pablo de Olavide, Sevilla, Spain; 7 Laboratorio de Biofilms Microbianos, CINDEFI-UNLP-CONICET, CCT La Plata, Facultad de Ciencias Exactas, Universidad Nacional de La Plata, Buenos Aires, Argentina; Universidad Andres Bello, Santiago, Chile

**Keywords:** experimental evolution, metabolic adaption, *Galleria *infection, cyclic GMP, biofilm, genomic analyses

## Abstract

Bacterial adaptation to stress can lead to phenotypic variants with diverse levels of niche competitiveness, pathogenicity, and antimicrobial resistance. In this work, we employed experimental evolution to investigate whether exposure to various stress conditions results in new phenotypic and metabolic properties in clinical and environmental strains of *Vibrio cholerae*. Our findings revealed the emergence of variants with metabolic and genetic variations and enhanced survival under stress compared to the parental isolates. Phenotypic changes in the evolved variants included colony morphology, biofilm formation, and the appearance of proteolytic and hemolytic activities. The variants demonstrated metabolic changes in the preferred use of carbon, nitrogen, phosphorous, and sulfur substrates, while the genetic changes included single nucleotide polymorphisms (SNPs), breakpoints, translocations, and single nucleotide insertions and deletions. Mutations in genes encoding EAL and HD-GYP domain-containing proteins correlated with increased biofilm formation and different colony morphotypes. The combined analysis of the metabolic and genomic data pointed to pathways implicated in stress survival. The environmental strains were generally more pathogenic than the clinical strains in the *Galleria mellonella* infection model prior to the experimental evolution, and these differences did not change in the evolved variants. This study highlights the contribution of stress conditions as drivers for the evolution of genetic modifications and metabolic adaptation in *V. cholerae*, which may explain the continuous evolution of El-Tor biotype strains toward variants with improved survival in the environment.

**IMPORTANCE:**

How *Vibrio cholerae*, the causative agent of cholera, survives during the periods between outbreaks remains a critical question. Using experimental evolution based on serial bacterial passages in culture media mimicking diverse environmental stress conditions, we investigated whether clinical and environmental isolates of *V. cholerae* develop changes in survival and in their metabolism. The evolved variants exhibited alterations in colony morphology, biofilm formation, and metabolism, including changes in the preferred use of carbon, nitrogen, phosphorous, and sulfur substrates. These changes were accompanied by various genetic modifications, notably in genes encoding second messenger molecules that regulate multiple biochemical pathways implicated in stress survival and increased pathogenic potential. Our results suggest a continuous evolution of *V. cholerae* strains toward variants displaying increased survival under environmental stress conditions that may also be encountered in the human host.

## INTRODUCTION

Cholera, an infectious disease caused by *Vibrio cholerae*, remains a significant global health threat, particularly in developing countries (1, 2). The disease displays a characteristic pattern of intermittent seasonal outbreaks (3, 4). Presumably, *V. cholerae* is exposed to multiple environmental stress cues arising from survival in biotic and abiotic environments and adaptation to human and non-human hosts. Diverse stress factors provide selective pressures favoring the rise of bacterial variants with varying levels of metabolic adaptation, pathogenicity, ecological competitiveness, and antimicrobial resistance (5–10).

Variants may exhibit phenotypic changes in motility, biofilm formation, chemotaxis, virulence factor expression, proteolysis, hemolysis, colony morphology changes (11, 12), and metabolic versatility such as the differential utilization of available substrates (13). These phenotypes usually result from underlying genetic variations and may include enhanced pathogenicity (14). Complex and often highly interconnected regulatory networks underpin phenotypic changes in bacteria, allowing for environmental persistence and host adaptation (15–17). For example, a mutation of *mucA* in *Pseudomonas aeruginosa* yielded mucoid colonies with improved capacity to colonize the lungs (18). In *V. cholerae*, deletions in *vacJ-ccmH mlaF, dacB*, and *ihfA* genes rescued motility (19), while deletion of *mutS* yielded wrinkled colonies with robust biofilm formation and colonization ability (20).

Despite an improved understanding of its epidemiology, cholera in Ghana represents a major public health challenge among many poor and vulnerable communities (21). We used an experimental evolution approach to evaluate whether *in vitro* exposure to multiple stress conditions results in improved survival and metabolic reprograming in clinical and environmental strains of *V. cholerae* isolated in Ghana. While phenotypic and genetic drivers of bacterial variants can be investigated by experimental evolution under controlled exposure to stress conditions (22), the methodology has only been used in a few cases to examine *V. cholerae* (19, 23, 24). Our findings indicate that specific phenotypic changes and genomic alterations likely influence the survival of the evolved variants under diverse stress conditions and different substrate utilization. By integrating with genomic data, we derived metabolic pathways implicated in stress survival and potentially also in microbial virulence.

## RESULTS

### Evolved variants reveal multiple phenotypic variations

**Fig 1:**
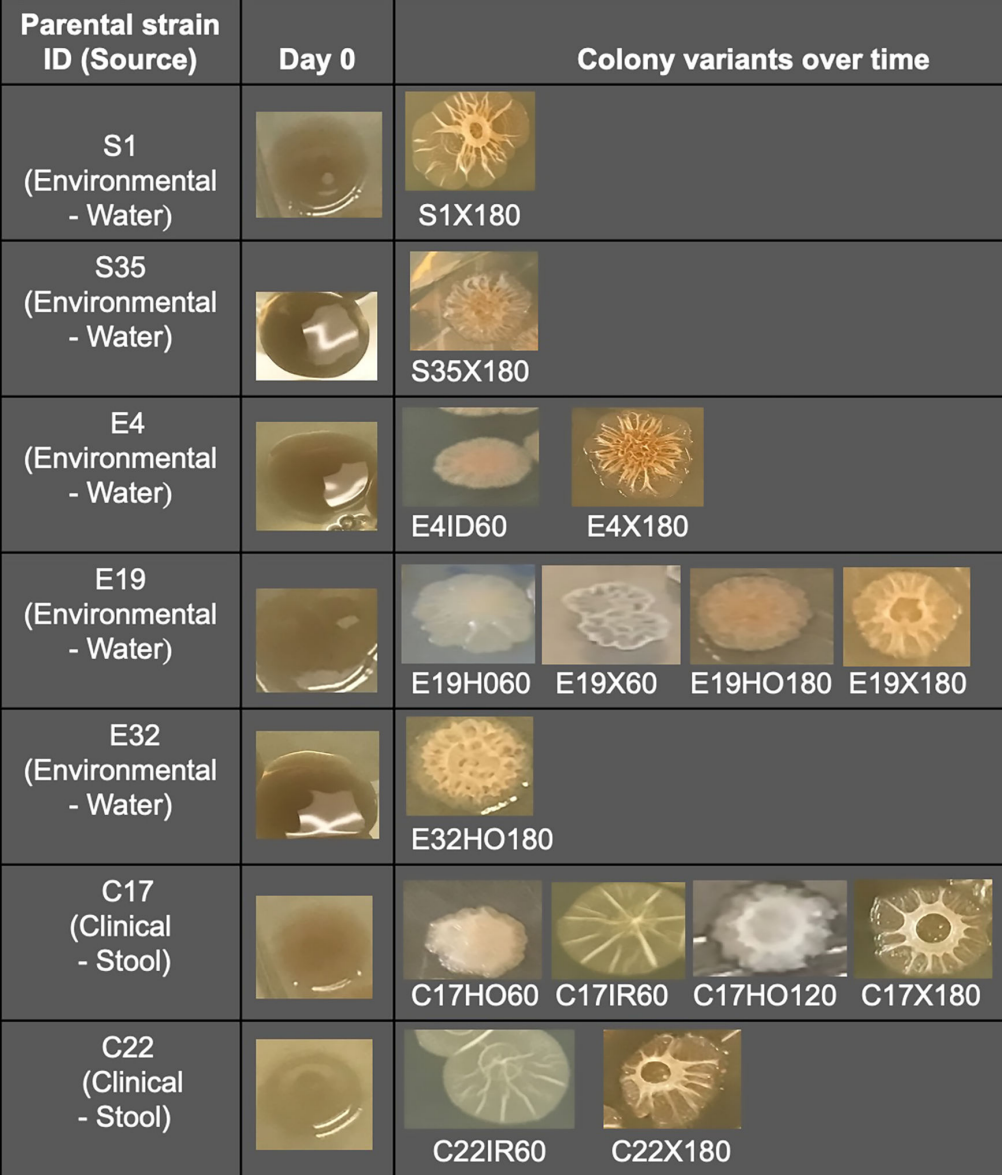
Colony variants observed at different harvest times compared to parental strains of the *V. cholerae* isolates. The colony morphologies of the LB negative control remain stable over the course of the experiment. The variants were labeled by combining the strain designation, stress conditions (X, oxidative stress; ID, iron depleted; HO, osmotic stress; IR, iron replete), and harvest time in days.

We employed experimental evolution on our set of environmental and clinical *V. cholerae* strains isolated in Ghana to identify survival and metabolic adaptive phenotypes arising from bacterial exposure to diverse stress conditions (Fig. S1). These conditions included iron excess, iron limitation, low pH, oxidative, and osmotic stress, all of which mimic both the gut environment, in part, and environmental conditions outside the human host. Growth in lysogeny broth (LB) medium was used as a control. Despite the presence of colonies of identical morphology to the parental strains at day 0, the evolved variants produced different colony morphotypes at various harvest times, especially after day 60 (Fig. 1). Novel colony morphotypes were mostly observed with evolved variants under oxidative and osmotic stress. Biofilm mass formation was an unstable phenotype, either increasing or decreasing over time and stress conditions without a defined pattern, except for isolates E32 and C22, where biofilm mass remained stably increased, relative to time 0 (Table 1; Fig. S2). In contrast, β**-**hemolysis production showed an increasing trend over time in most of the evolved isolates (Table 1; Fig. S3). Compared with the parental strains at day 0, protease activity exhibited a generally decreasing trend in the various passages following stress exposure (Table 1; Fig. S4). Variants that remained viable at their final harvest timepoint and exhibited increased biofilm formation, proteolytic and β**-**hemolytic activities, and changes in colony morphotypes (Table 1; Fig. 1) were selected for comparative phenotypic array analysis based on substrate utilization and *Galleria mellonella* infection assays.

**TABLE 1:** Biofilm formation, and protease and hemolytic activities of selected *V. cholerae* variants compared to their parental strains at day 0^*a*^

Parental strain ID	Evolved variant	Accession number	Biofilm formation	Protease activity	Hemolytic activity
S1 (day 0)		SAMN26804320			
	**T1**(S1HO180)	SAMN27544053	Decreased	Decreased	Increased
	**T2**(S1NLB180)	SAMN27544054	Decreased	Decreased	Increased
	**T3**(S1ID200)	SAMN27544055	No change	Decreased	Increased
	**T4**(S1X180)	SAMN27544056	Increased	Decreased	Increased
	**T5**(S1IR60)	SAMN27544057	Increased	No change	Increased
	**T6**(S1NLB120)	SAMN27544058	Increased	Decreased	Increased
	**T7**(S1IR200)	SAMN27544059	No change	Decreased	Increased
S35 (day 0)		SAMN26804321			
	**T9**(S35HO180)	SAMN27544060	Decreased	Decreased	Increased
	**T10**(S35X180)	SAMN27544061	Increased	Decreased	Increased
	**T41**(S35ID200)	SAMN27544090	Decreased	Decreased	Increased
E4 (day 0)		SAMN26804322			
	**T11**(E4ID60)	SAMN27544062	Increased	Decreased	Increased
	**T12**(E4ID200)	SAMN27544063	Decreased	Decreased	Increased
	**T13**(E4X180)	SAMN27544064	Increased	Decreased	Increased
	**T14**(E4PH150)	SAMN27544065	Decreased	Decreased	Increased
	**T15**(E4HO180)	SAMN27544066	Decreased	Decreased	Increased
	**T16**(E4NLB180)	SAMN27544067	Decreased	Decreased	Increased
	**T18**(E4IR200)	SAMN27544068	Decreased	Decreased	Increased
E19 (day 0)		SAMN26804323			
	**T19**(E19NLB180)	SAMN27544069	Decreased	Decreased	Increased
	**T20**(E19X180)	SAMN27544070	Increased	Decreased	Increased
	**T21**(E19HO180)	SAMN27544071	Increased	Decreased	Increased
	**T22**(E19ID200)	SAMN27544072	Increased	Decreased	Decreased
E30 (day 0)		SAMN26804324			
	**T23**(E30HO60)	SAMN27544073	Decreased	Increased	No change
	**T24**(E30PH112)	SAMN27544074	Decreased	Increased	Decreased
E32 (day 0)		SAMN26804325			
	**T25**(E32HO120)	SAMN27544075	Increased	Decreased	Increased
	**T26**(E32HO180)	SAMN27544076	Increased	Decreased	Increased
C6 (day 0)		SAMN26804327			
	**T27**(C6HO180)	SAMN27544077	Increased	Decreased	Increased
	**T28**(C6NLB180)	SAMN27544078	Decreased	Decreased	Increased
	**T29**(C6IR200)	SAMN27544079	Increased	Decreased	No change
C17 (day 0)		SAMN26804329			
	**T30**(C17IR60)	SAMN27544080	Increased	No change	Decreased
	**T31**(C17NLB180)	SAMN27544081	Increased	Decreased	Decreased
	**T32**(C17HO180)	SAMN27544082	Decreased	Increased	Increased
	**T33**(C17X180)	SAMN27544083	Increased	Decreased	Increased
	**T34**(C17ID200)	SAMN27544084	No change	Decreased	Increased
	**T35**(C17IR200)	SAMN27544085	Decreased	Decreased	Increased
C22 (day 0)		SAMN26804330			
	**T36**(C22HO180)	SAMN27544086	Increased	Decreased	Increased
	**T37**(C22ID200)	SAMN27544087	Increased	Decreased	Increased
	**T38**(C22X180)	SAMN27544088	Increased	Decreased	Increased
	**T39**(C22IR200	SAMN27544089	Increased	Decreased	No change

^*a*^ The evolved variants were labeled combining the strain designation, stress conditions (X, oxidative stress; ID, iron depleted; HO, osmotic stress; IR, iron replete; NLB, just LB), and harvest time in days. The T designation represents the strain number assigned to each variant to facilitate the downstream analyses.

### Genomic analyses of evolved variants demonstrate numerous genetic changes compared with the parental strains

**Fig 2:**
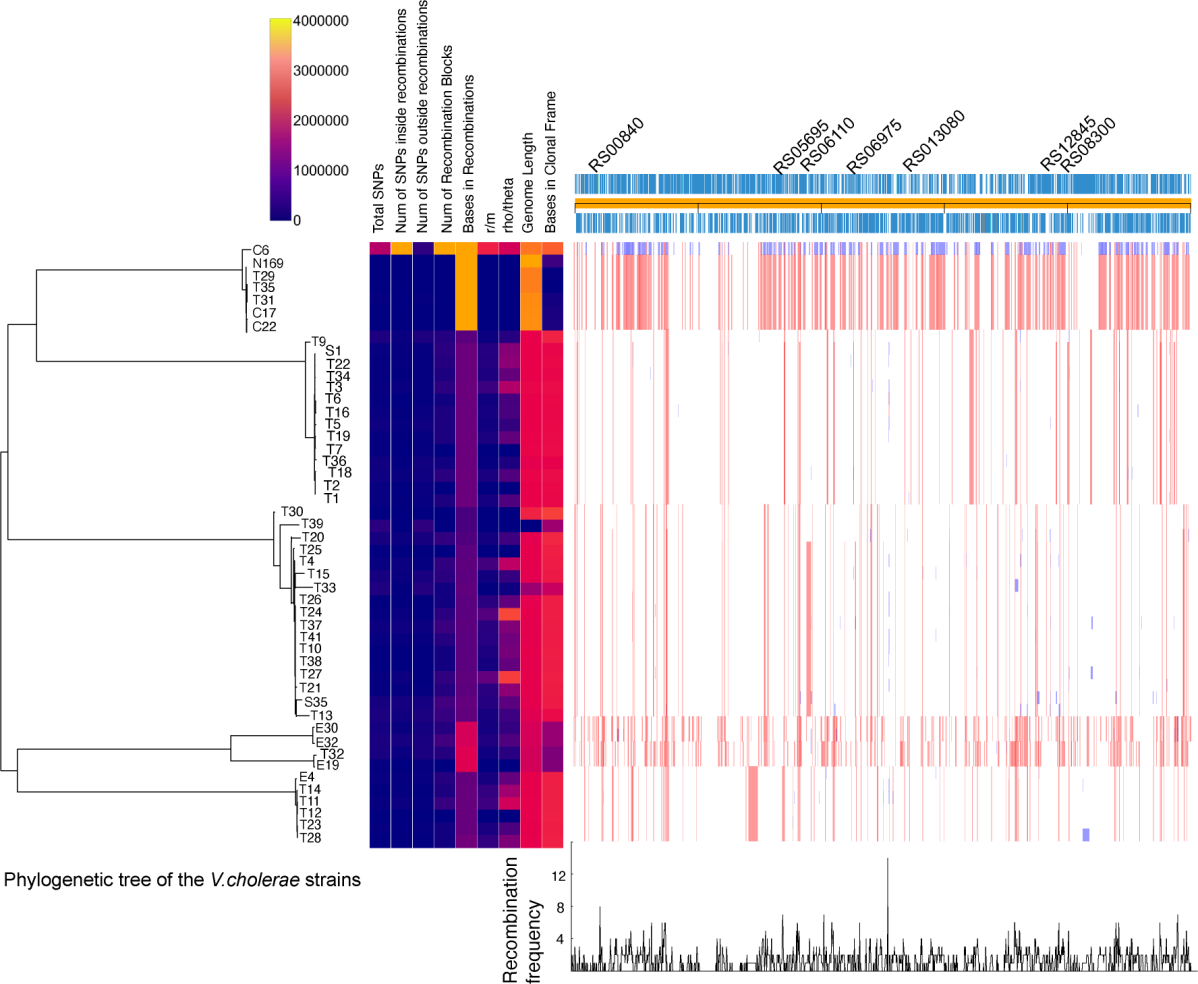
Homologous recombination events detected in the *V. cholerae* strains and evolved variants. Phandango plot of recombination regions detected with Gubbins using *V. cholerae* O1 biovar El Tor strain N16961 as the reference genome. A phylogenetic tree was generated from the number of SNPs and grouped into six clusters. The homologous recombination events are shown by blocks; blue-colored blocks are unique events in strains/evolved variants and red-colored blocks are multiple events in strains/evolved variants. The genes found in the recombination hotspots are marked at the top of the plot and are calculated based on the identification of genomic regions that demonstrate higher levels of recombination compared to the rest of the genome. The recombination frequency (y-axis) with peaks indicates the region of the recombination hotspot. Total SNPs: total number of base substitutions reconstructed onto the branch. Num of SNPs inside recombinations: number of base substitutions reconstructed onto the branch that falls within predicted recombination (**R**). Num of SNPs outside recombinations: number of base substitutions reconstructed onto the branch that falls outside of predicted recombination, i.e., predicted to have arisen by point mutation(m). Num of recombination blocks: total number of recombination blocks reconstructed onto the branch. Bases in recombinations: total length of all recombination events reconstructed onto the branch. r/m: the ratio of recombination to mutation ratio value for the branch. This value gives a measure of the relative impact of recombination versus mutation on the genetic variation accumulated on the branch. rho/theta: the ratio of the number of recombination events to point mutations on a branch; a measure of the relative rates of recombination and point mutation across the entire population. Genome length: the total number of aligned bases between the ancestral and descendent nodes for the branch excluding any missing data or gaps in either. The bases in the clonal frame refer to the proportion of the genome that has not been affected by recombination and retains the signal of clonal inheritance.

All the strains were characterized by whole genome sequencing (Table 2). The genome sizes of the parental strains and evolved variants ranged from 3.9 to 4.45 Mbp. Comparative genomic analyses were done to understand the underlying genetic changes responsible for the survival and newly acquired phenotypic characteristics of the evolved *V. cholerae* strains. We reconstructed short-term bacterial evolution in our isolates by iteratively identifying loci containing an elevated density of base substitutions, which provide recombination hotspots, and concurrently constructing a phylogeny based on the putative point mutations outside of these regions (25). Compared with parental strains, the evolved variants had numerous single nucleotide polymorphisms (SNPs), breakpoints, translocations, and single nucleotide insertions and deletion mutations (Table 2). Recombination analysis by genome-wide alignment of the parental strains and evolved variants around their origin of replication revealed recombination hotspots, especially in genes *RS00840, RS06110, RS06975,* and *RS12845* (Fig. 2), conserved in all strains, which encode a GGDEF domain-containing protein, a response regulator transcription factor, a cadmium-translocating P-type ATPase and an oligosaccharide flippase family protein, respectively.

**TABLE 2:** Summary of genomic differences between the parental strains and the evolved variants

*V. cholerae* strains/variants	Genome size (Mbp)	Breakpoints	Relocations^*a*^	Translocations^*a*^	Inversions	Major insertions^*b*^	Total SNPs	Total indels^*c*^
**S1 (parental**)	3.98							
T1(S1HO180)	3.98	96	0	9	0	43	245	29
T2(S1NLB180)	3.98	166	0	22	0	39	265	29
T3(S1ID200)	3.97	152	1	16	0	25	25	18
T4(S1X180)	3.92	1,293	12	80	0	442	54,113	3,031
T5(S1IR60)	3.97	117	0	14	0	30	31	6
T6(S1NLB120)	3.98	117	0	15	0	34	124	19
T7(S1IR200)	3.98	78	0	7	0	26	119	35
**S35 (parental**)	3.92							
T9(S35HO180)	3.97	1,271	11	67	0	428	54,184	3,057
T10(S35X180)	3.92	107	0	12	0	43	16	5
T41(S35ID200)	3.92	151	1	18	0	43	18	11
**E4 (parental**)	4.05							
T11(E4ID60)	4.05	111	1	16	0	428	54,184	3,057
T12(E4ID200)	4.05	127	2	18	0	38	16	5
T13(E4X180)	4.05	111	1	16	0	40	15	4
T14(E4PH150)	4.05	127	2	18	0	40	15	4
T15(E4HO180)	3.92	1,350	31	65	0	457	52,641	3,024
T16(E4NLB180)	4	1,435	20	77	0	519	55,000	3,141
T18(E4IR200)	3.98	1,439	23	71	0	455	54,966	3,130
**E19 (parental**)	3.97							
T19(E19NLB180)	3.97	1,312	12	60	0	513	61,571	3,990
T20(E19X180)	3.91	1,289	21	66	0	540	61,571	3,990
T21(E19HO180)	3.92	1,283	24	64	0	532	61,051	3,904
T22(E19ID200)	3.97	1,307	14	58	0	514	61,273	3,995
**E30 (parental**)	3.97							
T23(E30HO60)	4.06	1,397	13	67	0	667	63,439	4,236
T24(E30PH112)	3.92	1,191	15	59	0	526	62,663	3,698
**E32 (parental**)	3.97							
T25(E32HO120)	3.92	1,207	34	29	3	565	62,694	3,725
T26(E32HO180)	3.92	1,201	35	27	3	558	62,554	3,736
**C6 (parental**)	4.04							
T27(C6HO180)	3.92	1,247	16	70	0	527	55,085	3,420
T28(C6NLB180)	4.06	1,357	10	93	0	618	56,348	3,532
T29(C6IR200)	4.01	624	4	49	0	191	22,047	1,551
**C17 (parental**)	4.09							
T30(C17IR60)	4.45	1,143	2	46	0	637	52,645	2,994
T31(C17NLB180)	4.1	98	0	14	0	65	86	16
T32(C17HO180)	3.97	1,129	5	67	0	424	63,713	4,330
T33(C17X180)	3.91	1,170	6	74	0	459	55,033	3,066
T34(C17ID200)	3.97	1,146	9	71	0	44	157	93
T35(C17IR200)	4.01	172	0	17	0	44	157	93
**C22 (parental**)	4.09							
T36(C22HO180)	3.98	1,114	11	79	0	438	53,735	2,895
T37(C22ID200)	3.91	1,148	8	79	0	463	55,037	3,177
T38(C22X180)	3.92	1,170	7	70	0	481	54,969	3,188
T39(C22IR200)	3.97	1,086	15	73	0	405	53,719	2,839

^*a*^ Relocations and translocations refer to different types of chromosomal rearrangements. Relocations involve the movement of segments of DNA to a new position within the same genome which includes duplications, or the movement of genes or segments within the same chromosome. Translocations involve the rearrangement of parts between non-homologous chromosomes which includes the transfer of genetic material between different genomic regions, or the incorporation of DNA from external sources into the genome, resulting in new sequences appearing at different loci than their original locations.

^*b*^ Insertions large enough to break an alignment.

^*c*^ Single nucleotide insertions/deletions.

**Fig 3:**
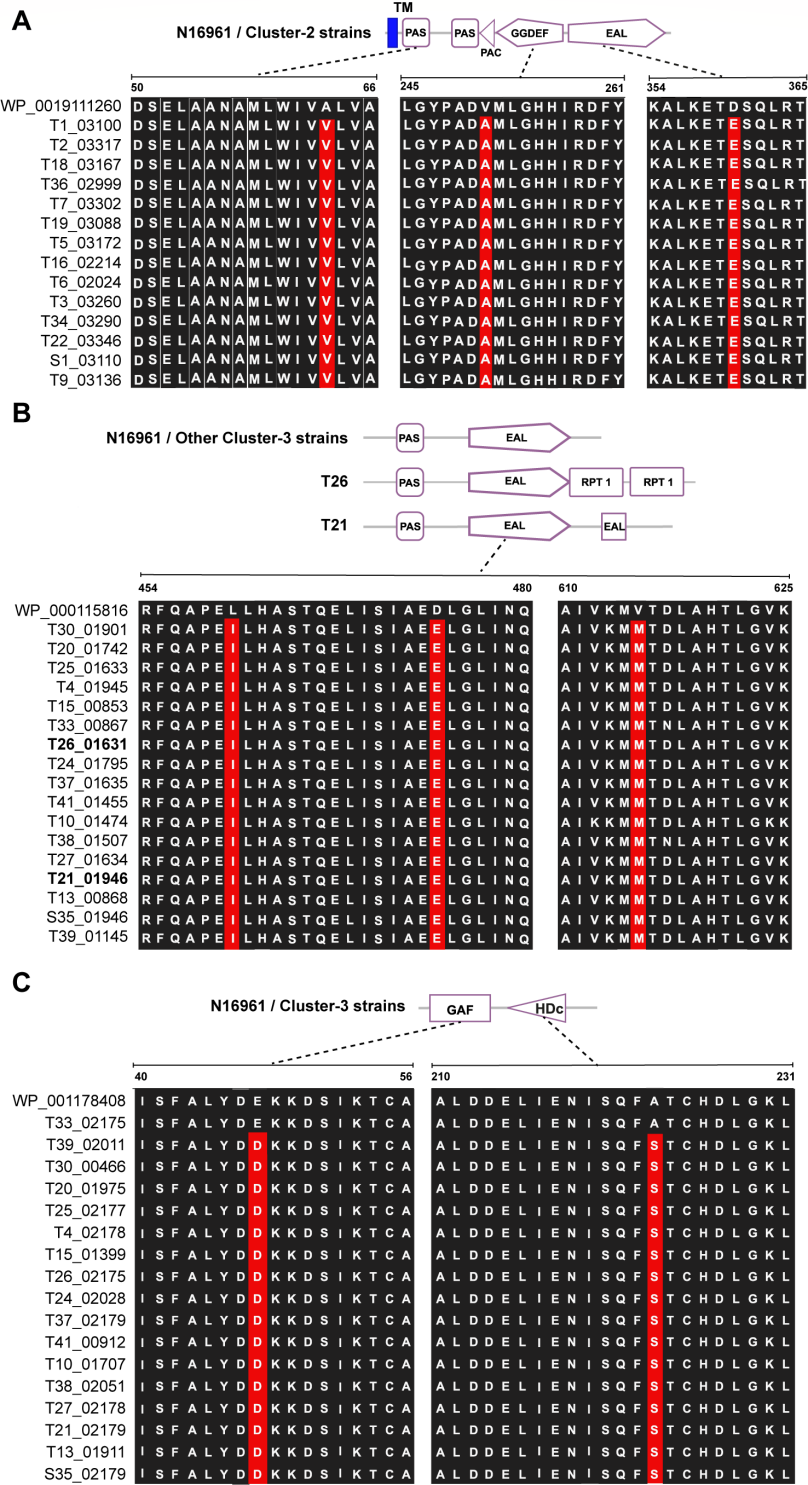
Domain architectures of the EAL-domain containing proteins of the strains in clusters 2 and 3 compared to the reference *V. cholerae* strain El Tor strain N16961. Only alignments of domains with amino acid replacements are shown. (**A**) Domain architecture of the EAL-domain containing proteins of cluster-2 strains compared to the EAL domain-containing protein WP0019111260 of N16961. (**B**) Domain architecture of the EAL-domain containing proteins of cluster-3 strains compared to the protein WP00115816 of N16961. (**C**) Domain architecture of the GAF/Hdc domain-containing proteins from cluster-3 strains compared to WP001178408 of N1691. Amino acid replacements are indicated in red background.

Relative to the *V. cholerae* El-Tor N16961 reference genome, all the strains and variants clustered into six phylogenetic groups (Fig. 2). Most of the evolved variants were in clusters 2 and 3 (Fig. 2). Variants in cluster 3 included those with increased biofilm formation (Table 1; Fig. S2) and exhibited different colony morphotypes compared to the parental strain (Fig. 1), while the variants in cluster 2 showed variable biofilm formation (Table 1; Fig. S2) and identical colony morphologies with the parental strains. Unique SNPs against the reference genome were identified in all the clusters, including 214, 20, 12, 43, 60, and 11 genes with SNPs for clusters 1 through 6, respectively (Supplementary Data 1). Apart from other mutations, the two major clusters 2 and 3 strains had mutations in genes homologous to *N16961_RS00345* and *N16961_RS05910*, which encode EAL domain-containing proteins (Supplementary Data 1). Cluster 3 also had mutations in the HD-GYP domain-containing protein gene homologous to *N16961_RS02845* (Supplementary Data 1). Mutations were identified in the EAL and HD-GYP domains and in the sensory domains PAS, PAC, GGDEF, and GAF (Fig. 3A through C) of the encoded proteins. All proteins from cluster-3 strains were similar in domain architecture with the WP_000115816 of the N16961 reference strain, except for strains T26 and T21. The protein found in T26 also contained two Repeat domains (RPT1) that mediate protein-protein interactions (26), while the protein in T21 contains a second, truncated EAL domain (Fig. 3B). Since all these proteins are involved in the synthesis and turnover of cyclic-di-GMP (c-di-GMP), a second messenger regulating multiple genes associated with bacterial stress adaptation (27), our results suggest that the levels of c-di-GMP are an important component underlying the phenotypic variations observed in the evolved strains.

### Substrate utilization profile analyses indicate differences between the parental strains and evolved variants

**Fig 4:**
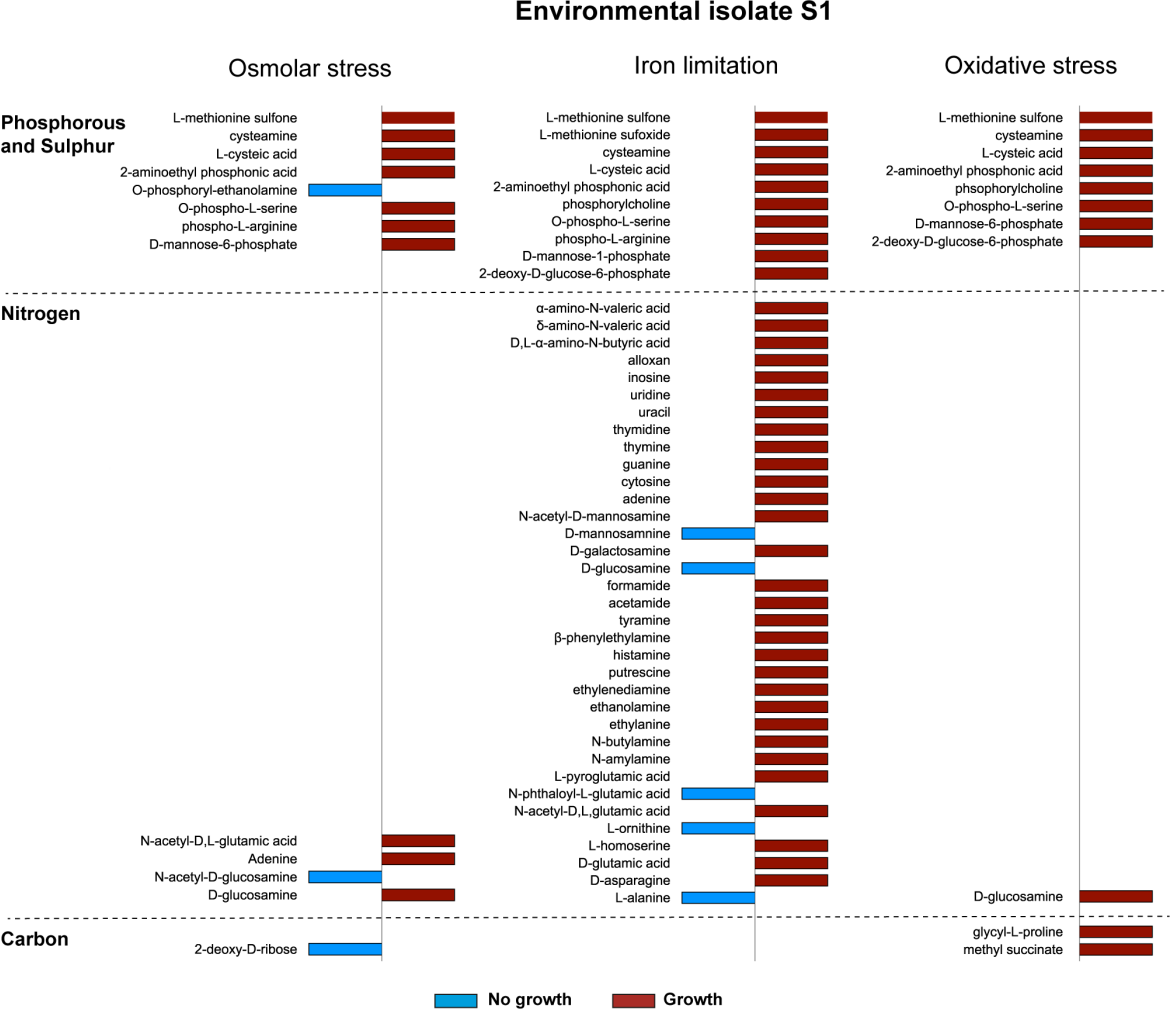
Unique substrate utilization profiles (carbon, nitrogen, phosphorous, and sulfur) of the environmental isolate S1 variants that evolved under osmolar stress, iron limitation, and oxidative stress, which were not present in S1 grown in LB. The complete set of unfiltered results is shown in Fig. S5 to S7.

**Fig 5:**
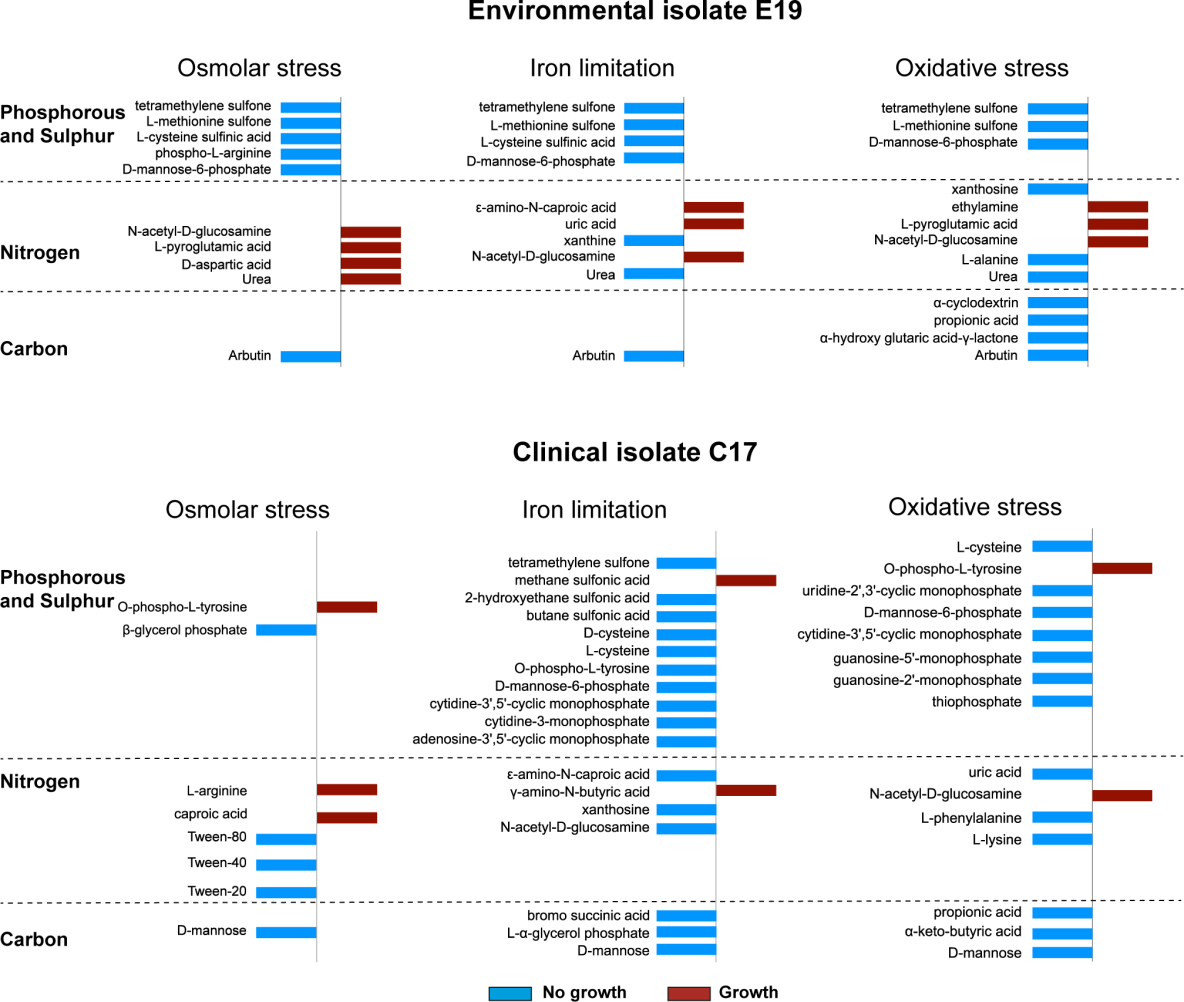
Unique substrate utilization profiles (carbon, nitrogen, phosphorous, and sulfur) of the environmental isolates E19 and C17 variants that evolved under osmolar stress, iron limitation, and oxidative stress, which were not present in the respective E19 and C17 strains grown in LB. The complete set of unfiltered results is shown in Fig. S5 to S7.

To determine metabolic phenotypes, a subset composed of two environmental strains (S1 and E19) and one clinical strain (C17) was selected based on the long-term viability (180 and 200 days, respectively) of their respective evolved variants in similar stress conditions (osmolar and oxidation stress and iron limitation) and with changes in one or more of the phenotypes associated with biofilm production and exoprotein activities (Table 1). To pinpoint unique variations in substrate utilization profiles, the results were compared relative to the isolates evolved in LB, which served as a negative control (see complete unfiltered data set in Fig. S5 to S7). For the isolate S1, evolved variants under osmolar, iron limitation and oxidative stress displayed increased growth under phosphorous and sulfur sources, especially in relation to cysteine, methionine, arginine, and phosphorylated sugars, suggesting a redirection of metabolic fluxes via the Krebs cycle (Fig. 4). Under iron limitation, the S1 isolate also displayed growth under multiple nitrogen sources, suggesting an increased metabolism geared to protein and the synthesis of nucleic acids. In contrast, the evolved variants of environmental isolate E19 displayed growth in some nitrogen sources when evolved under osmolar, iron limitation, and oxidative stress, in which the LB-grown counterparts could not grow (Fig. 5). Stress-evolved variants of E19 did not grow in the same phosphorous and sulfur sources employed by S1 variants (Fig. 5), suggesting differences in the manner that the two isolates responded to stress. Isolate E19 was the only one showing lack of growth in the presence of the β-glucoside arbutin under the three stress conditions examined (Fig. 5). The catabolism of β-glucosides in other bacteria depends on the activation of a strongly repressed cryptic aromatic β-glucoside utilization operon (*bglGFB*), which is only activated by mutations selected when bacteria are grown with β-glucoside as carbon sources (28). Since arbutin was utilized when E19 had evolved in LB, we speculate that the catabolism of β-glucosides in E19 variants is under negative regulation in the stress conditions examined. The evolved variants of the clinical isolate C17 had similar metabolic profiles to those of E19, but with more limitations to grow in phosphate/sulfur and some nitrogen sources under iron limitation (Fig. 5). Notably, the C17 variant evolved under osmolar stress did not grow in the presence of various forms of the polysorbate surfactant agent Tween, in agreement with studies indicating polysorbates can alter the bacterial cell envelope permeability (29).

We also aimed to correlate the metabolic changes in these isolates and variants by SNP analyses. As with the metabolic profiling, unique SNPs were investigated relative to the isolates evolved in LB, which served as a reference for the SNP analysis. Under osmolar stress, the S1 variant showed a SNP in the *hscA* gene, which encodes a homolog of Hsp70 that regulates the ATP-dependent transfer of FeS clusters to acceptor proteins (30) and also SNPs in ferredoxin and bacterioferritin encoding genes (Table 3). These results are consistent with the ability of this variant to grow under phosphorous and sulfur sources (Fig. 4), suggesting the mutations in these genes are beneficial to the bacterium. In contrast, E19 and C17 variants had a different set of SNPs, many of which encoded regulatory proteins and proteins involved in peptide transport, consistent with adaptation to osmolar stress (Table 3).

**TABLE 3:** Unique SNPs found in isolates S1, E19, and C17 at 180 days under osmolar stress

Gene	Product	Type of mutation ^*a*^		
		**S1**	**E19**	**C17**
*16364_S1_00310*	Hypothetical protein	SNP	NONE	NONE
*hscA_2*	Chaperone; homolog of Hsp70 that regulates the ATP-dependent transfer of FeS clusters to acceptor proteins	SNP	NONE	NONE
*rhtB_1*	Homoserine/homoserine lactone efflux protein	SNP	NONE	NONE
*16364_S1_03028*	Ferredoxin 1	SNP	NONE	NONE
*bfr*	Bacterioferritin	SNP	NONE	NONE
*16364_S1_00716*	Hypothetical protein	INS	NONE	NONE
*prt*	Microbial collagenase	SNP	NONE	NONE
*fldA*	Flavodoxin	SNP	NONE	NONE
*16364_S1_01470*	Hypothetical protein	INS	NONE	NONE
*fliM*	Flagellar motor switch protein	SNP	NONE	NONE
*16364_S1_02023*	Hypothetical protein	SNP	NONE	NONE
*uhpC_2*	Membrane sensor protein	SNP	NONE	NONE
*rimO*	Ribosomal protein S12 methylthiotransferase	SNP	NONE	NONE
*16364_S1_03570*	16364_S1_03570	DEL	NONE	NONE
*16364_S1_03573*	16364_S1_03573	DEL	NONE	NONE
*chb*	N,N'-diacetylchitobiose	SNP	NONE	NONE
*rseP*	Regulator of sigma-E protease	SNP	NONE	NONE
*tsf*	Elongation factor Ts	SNP	NONE	NONE
*abgT_1*	p-Aminobenzoyl-glutamate transport protein	SNP	NONE	NONE
*16364_S1_02621*	Hypothetical protein	SNP	NONE	NONE
*tagA*	N-acetylglucosaminyldiphosphoundecaprenol N-acetyl-beta-D-mannosaminyltransferase	NONE	SNP	NONE
*cdgJ_2*	Cyclic di-GMP phosphodiesterase	NONE	SNP	SNP
*sasA_9*	Adaptive-response sensory-kinase	NONE	SNP	SNP
*caiA*	caiA	NONE	DEL	NONE
*galS*	galS	NONE	DEL	NONE
*dcuA*	Anaerobic C4-dicarboxylate transporter	NONE	DEL	NONE
*btsS*	Sensor histidine kinase	NONE	SNP	NONE
*luxR*	HTH-type transcriptional regulator	NONE	INS	DEL
*[34701_S35_03533]*	[34701_S35_03533]	NONE	DEL	NONE
*pdeN*	Putative cyclic di-GMP phosphodiesterase	NONE	SNP	NONE
*34701_S35_03534*	34701_S35_03534	NONE	DEL	NONE
*bamE*	Outer membrane protein assembly factor	NONE	NONE	SNP
*dnaK_1*	Chaperone protein	NONE	NONE	SNP
*purL*	Phosphoribosylformylglycinamidine synthase	NONE	NONE	SNP
*34698_E19_00047*	Hypothetical protein	NONE	NONE	SNP
*metN*	Methionine import ATP-binding protein	NONE	NONE	SNP
*34698_E19_00076*	Hypothetical protein	NONE	NONE	SNP
*wcaJ*	UDP-glucose:undecaprenyl-phosphate glucose-1-phosphate transferase	NONE	NONE	INS
*mrdB*	Peptidoglycan glycosyltransferase	NONE	NONE	SNP
*mrdA*	Peptidoglycan D,D-transpeptidase	NONE	NONE	SNP
*rlmH*	Ribosomal RNA large subunit methyltransferase H	NONE	NONE	SNP
*lnt*	Apolipoprotein N-acyltransferase	NONE	NONE	SNP
*uvrB*	UvrABC system protein B	NONE	NONE	SNP
*luxO_1*	Regulatory protein	NONE	NONE	SNP
*moaA*	GTP 3',8-cyclase	NONE	NONE	SNP
*moaC*	Cyclic pyranopterin monophosphate synthase	NONE	NONE	SNP
*sohB*	Putative protease	NONE	NONE	SNP
*ackA_1*	Acetate kinase	NONE	NONE	SNP
*34698_E19_00294*	Hypothetical protein	NONE	NONE	DEL
*trpB*	Tryptophan synthase beta chain	NONE	NONE	SNP
*34698_E19_00317*	Hypothetical protein	NONE	NONE	SNP
*tcyP*	L-cystine uptake protein	NONE	NONE	SNP
*gyrA*	DNA gyrase subunit A	NONE	NONE	SNP
*dctM_1*	C4-dicarboxylate TRAP transporter large permease protein	NONE	NONE	SNP
*34698_E19_00491*	Hypothetical protein	NONE	NONE	SNP
*34698_E19_00607*	Hypothetical protein	NONE	NONE	SNP
*nagK_1*	N-acetyl-D-glucosamine kinase	NONE	NONE	SNP
*34698_E19_00618*	Hypothetical protein	NONE	NONE	SNP
*katG*	Catalase-peroxidase	NONE	NONE	SNP
*appB*	Cytochrome bd-II ubiquinol oxidase subunit 2	NONE	NONE	SNP
*budB*	Acetolactate synthase, catabolic	NONE	NONE	SNP
*34698_E19_02211*	Hypothetical protein	NONE	NONE	SNP
*rplY*	50S ribosomal protein L25	NONE	NONE	SNP
*34698_E19_02230*	Hypothetical protein	NONE	NONE	SNP
*ybhS*	Putative multidrug ABC transporter permease	NONE	NONE	SNP
*malK_2*	Maltose/maltodextrin import ATP-binding protein	NONE	NONE	SNP
*sapA*	Peptide transport periplasmic protein	NONE	NONE	SNP
*sapC*	Peptide transport system permease protein	NONE	NONE	SNP
*focA*	Putative formate transporter 1	NONE	NONE	SNP
*34698_E19_03563*	34698_E19_03563	NONE	NONE	DEL
*[34698_E19_03564]*	[34698_E19_03564]	NONE	NONE	DEL
*34698_E19_03565*	34698_E19_03565	NONE	NONE	DEL
*34698_E19_03566*	34698_E19_03566	NONE	NONE	DEL
*34698_E19_02343*	Hypothetical protein	NONE	NONE	SNP
*34698_E19_02359*	Hypothetical protein	NONE	NONE	SNP
*dctD_3*	C4-dicarboxylate transports transcriptional regulatory protein	NONE	NONE	SNP
*ulaA*	Ascorbate-specific PTS system EIIC component	NONE	NONE	SNP
*mtr*	Tryptophan-specific transport protein	NONE	NONE	SNP
*34698_E19_02415*	Hypothetical protein	NONE	NONE	SNP
*34698_E19_02447*	4-O-beta-D-mannosyl-D-glucose phosphorylase	NONE	NONE	SNP
*rapA_1*	RNA polymerase-associated protein	NONE	NONE	SNP
*tnsC*	Transposon Tn7 transposition protein	NONE	NONE	SNP
*pilT_2*	Twitching mobility protein	NONE	NONE	SNP
*yjjP*	Inner membrane protein	NONE	NONE	SNP
*34698_E19_02559*	Hypothetical protein	NONE	NONE	SNP
*uvrA*	UvrABC system protein A	NONE	NONE	SNP
*34698_E19_02569*	Hypothetical protein	NONE	NONE	SNP
*34698_E19_03567*	34698_E19_03567	NONE	NONE	DEL
*34698_E19_03568*	34698_E19_03568	NONE	NONE	DEL
*34698_E19_03569*	34698_E19_03569	NONE	NONE	DEL
*34698_E19_02575*	Hypothetical protein	NONE	NONE	SNP
*dxr*	1-Deoxy-D-xylulose 5-phosphate reductoisomerase	NONE	NONE	SNP
*34698_E19_02614*	Hypothetical protein	NONE	NONE	SNP
*34698_E19_02655*	Hypothetical protein	NONE	NONE	SNP
*flaD_3*	Flagellin D	NONE	NONE	SNP
*34698_E19_03570*	Hypothetical protein	NONE	NONE	DEL
*34698_E19_03571*	Hypothetical protein	NONE	NONE	DEL
*rplB*	50S ribosomal protein L2	NONE	NONE	SNP
*msrA*	Peptide methionine sulfoxide reductase	NONE	NONE	SNP
*tamA*	Translocation and assembly module subunit	NONE	NONE	SNP
*ubiX/thiB*	Flavin prenyltransferase UbiX/Thiamine-binding periplasmic protein	NONE	NONE	SUB
*34698_E19_03572*	Hypothetical protein	NONE	NONE	DEL
*yfhL*	Ferredoxin	NONE	NONE	SNP
*ppx*	Exopolyphosphatase	NONE	NONE	SNP
*secD_2*	Protein translocase subunit	NONE	NONE	SNP
*34698_E19_02860*	Hypothetical protein	NONE	NONE	SNP
*citF*	Citrate lyase alpha chain	NONE	NONE	SNP
*34698_E19_02912*	UPF0394 membrane protein XF_0766	NONE	NONE	SNP
*tal/deoR*	Transaldolase/deoxyribonucleoside regulator	NONE	NONE	DEL
*purK*	N5-carboxyaminoimidazole ribonucleotide synthase	NONE	NONE	SNP
*trkI*	Trk system potassium uptake protein	NONE	NONE	SNP
*34698_E19_03005*	Hypothetical protein	NONE	NONE	SNP
*yihG*	Putative acyltransferase	NONE	NONE	SNP
*acuI*	Putative acrylyl-CoA reductase	NONE	NONE	SNP
*34698_E19_03050*	Hypothetical protein	NONE	NONE	SNP
*34698_E19_03050*	Hypothetical protein	NONE	NONE	SNP
*34698_E19_03112*	Hypothetical protein	NONE	NONE	SNP
*cusA*	Cation efflux system protein	NONE	NONE	SNP
*dacA_2*	D-alanyl-D-alanine carboxypeptidase	NONE	NONE	SNP
*birA*	Bifunctional ligase/repressor	NONE	NONE	SNP
*tusD*	Sulfurtransferase TusD	NONE	NONE	SNP
*34698_E19_00710*	Hypothetical protein	NONE	NONE	SNP
*34698_E19_00726*	Hypothetical protein	NONE	NONE	SNP
*lldP*	L-lactate permease	NONE	NONE	SNP
*argT_1*	Lysine/arginine/ornithine-binding periplasmic protein	NONE	NONE	SNP
*34698_E19_00748*	Hypothetical protein	NONE	NONE	DEL
*34698_E19_00838*	47 kDa outer membrane protein	NONE	NONE	SNP
*34698_E19_00841*	Hypothetical protein	NONE	NONE	SNP
*luxQ*	Autoinducer 2 sensor kinase/phosphatase	NONE	NONE	SNP
*34698_E19_00946*	Hypothetical protein	NONE	NONE	SNP
*34698_E19_00968*	Hypothetical protein	NONE	NONE	SNP
*uhpA*	Transcriptional regulatory protein	NONE	NONE	SNP
*vraR*	Response regulator protein	NONE	NONE	SNP
*34698_E19_03216*	Hypothetical protein	NONE	NONE	SNP
*34698_E19_03293*	Hypothetical protein	NONE	NONE	SNP
*34698_E19_03295*	Hypothetical protein	NONE	NONE	SNP
*34698_E19_03326*	Hypothetical protein	NONE	NONE	DEL
*ileS*	Isoleucine–tRNA ligase	NONE	NONE	SNP
*murQ_2*	N-acetylmuramic acid 6-phosphate etherase	NONE	NONE	SNP
*34698_E19_03362*	Hypothetical protein	NONE	NONE	SNP
*34698_E19_03421*	Hypothetical protein	NONE	NONE	SNP
*atpC*	ATP synthase epsilon chain	NONE	NONE	SNP
*gcvP*	Glycine dehydrogenase (decarboxylating)	NONE	NONE	SNP
*tehA*	Tellurite resistance protein	NONE	NONE	SNP
*34698_E19_01057*	Hypothetical protein	NONE	NONE	SNP
*34698_E19_01058*	Phosphoserine phosphatase	NONE	NONE	SNP
*arcB*	Aerobic respiration control sensor protein	NONE	NONE	SNP
*gltB_1*	Ferredoxin-dependent glutamate synthase 1	NONE	NONE	SNP
*gltB_2*	Glutamate synthase [NADPH] large chain	NONE	NONE	SNP
*murC*	UDP-N-acetylmuramate–L-alanine ligase	NONE	NONE	SNP
*ampD*	1,6-Anhydro-N-acetylmuramyl-L-alanine amidase	NONE	NONE	SNP
*nadC*	Nicotinate-nucleotide pyrophosphorylase [carboxylating]	NONE	NONE	SNP
*rlmD*	23S rRNA (uracil(1939)-C(5))-methyltransferase	NONE	NONE	SNP
*34698_E19_01190*	Hypothetical protein	NONE	NONE	SNP
*potE*	Putrescine transporter	NONE	NONE	SNP
*egtB*	Hercynine oxygenase	NONE	NONE	INS
*sdcS_2*	Sodium-dependent dicarboxylate transporter	NONE	NONE	SNP
*34698_E19_01315*	Cyclic di-GMP binding protein VCA0042	NONE	NONE	SNP
*34698_E19_01319*	Hypothetical protein	NONE	NONE	SNP
*ydgJ*	Putative oxidoreductase	NONE	NONE	INS
*34698_E19_01413*	Hypothetical protein	NONE	NONE	SNP
*asd2*	Aspartate-semialdehyde dehydrogenase 2	NONE	NONE	SNP
*sdhD*	Succinate dehydrogenase hydrophobic membrane anchor subunit	NONE	NONE	SNP
*sucA*	2-Oxoglutarate dehydrogenase E1 component	NONE	NONE	SNP
*rhaR_3*	HTH-type transcriptional activator	NONE	NONE	SNP
*34698_E19_01495*	Hypothetical protein	NONE	NONE	SNP
*34698_E19_01498*	Hypothetical protein	NONE	NONE	SNP
*ccmA*	Cytochrome c biogenesis ATP-binding export protein	NONE	NONE	SNP
*siaQ/siaP*	Sialic acid TRAP transporter small permease protein	NONE	NONE	SNP
*rcsC_6*	Sensor histidine kinase	NONE	NONE	SNP
*34698_E19_01640*	Hypothetical protein	NONE	NONE	SNP
*ycfP*	UPF0227 protein	NONE	NONE	SNP
*34698_E19_01698*	Hypothetical protein	NONE	NONE	SNP
*clpX/clpP*	ATP-dependent Clp protease ATP-binding subunit	NONE	NONE	SNP
*34698_E19_01732*	Hypothetical protein	NONE	NONE	SNP
*34698_E19_03559*	34698_E19_03559	NONE	NONE	DEL
*mutS*	DNA mismatch repair protein	NONE	NONE	DEL
*mrcB*	Penicillin-binding protein 1B	NONE	NONE	SNP
*oppF_3*	Oligopeptide transport ATP-binding protein	NONE	NONE	SNP
*34698_E19_01906*	Hypothetical protein	NONE	NONE	DEL
*edd*	Phosphogluconate dehydratase	NONE	NONE	SNP
*cadA*	Inducible lysine decarboxylase	NONE	NONE	SNP
*purD*	Phosphoribosylamine–glycine ligase	NONE	NONE	SNP
*34698_E19_02009*	Hypothetical protein/hypothetical protein	NONE	NONE	SNP
*34698_E19_02012*	Hypothetical protein	NONE	NONE	SNP
*34698_E19_02024*	Hypothetical protein	NONE	NONE	SNP
*rlmJ*	Ribosomal RNA large subunit methyltransferase J	NONE	NONE	SNP
*34698_E19_02109*	Hypothetical protein	NONE	NONE	SNP
*hslU*	ATP-dependent protease ATPase subunit	NONE	NONE	SNP
*cytR*	HTH-type transcriptional repressor	NONE	NONE	SNP
*spoT*	Guanosine-3',5'-bis(diphosphate) 3'-pyrophosphohydrolase	NONE	NONE	SNP
*34698_E19_02162*	Hypothetical protein/hypothetical protein	NONE	NONE	SNP
*34698_E19_03562*	Hypothetical protein	NONE	NONE	DEL

^*a*^ None, non-mutated gene; SNP, single nucleotide polymorphism resulting in a non-synonymous codon; DEL, mutation causing a complete or partial gene deletion. The raw data from Snippy used for this analysis are found in Supplementary Data 2.

Examination of SNPs in the variants evolved under iron limitation revealed common SNPs and deletions in the three isolates (Table 4), especially mutations in the *fur* iron regulator protein gene that potentially would relieve repression of the iron uptake systems under iron-limiting conditions. Moreover, all three isolates had mutations (SNPs or deletions) in the cyclic di-GMP phosphodiesterase encoding gene *cdgJ*, and in the *iscR* gene encoding a regulator involved in the synthesis of regulators which plays a role regulating bacterial behavior properties in *V. cholerae* including biofilm and motility (31, 32). These mutations could also be involved in other functions directly or indirectly modulating iron uptake. Typically, the *iscR* gene is implicated in the regulation of host-derived nitrosative stress and iron starvation, and the transition from viable but non-culturable to culturable states (33, 34). The S1 isolate was the only one with a mutation in the *barA* gene, which encodes a global regulator involved in controlling multiple genes during the stationary phase (35). This is consistent with the unique ability of this isolate to grow in multiple nitrogen sources (Fig. 4).

**TABLE 4:** Unique SNPs found in isolates S1, E19, and C17 at 180 days under iron limitation and oxidative stress

Gene	Product	Type of mutation ^*a*^		
		S1	E19	C17
**Iron limitation**				
*iscR*	HTH-type transcriptional regulator	INS	INS	INS
*acp*	Sodium/proton-dependent alanine carrier protein	SNP	SNP	SNP
*cdgJ_2*	Cyclic di-GMP phosphodiesterase	SNP	DEL	DEL
∆*16364_S1_00938‚16364_S1_00939*	Hypothetical proteins	DEL	DEL	DEL
*nhaA*	Na(+)/H(+) antiporter	SNP	NONE	NONE
*marC*	UPF0056 inner membrane protein	DEL	DEL	DEL
*fur*	Ferric uptake regulation protein	SNP	SNP	SNP
∆*16364_S1_03551‚16364_S1_03552*	Hypothetical proteins	DEL	NONE	NONE
*mepA_2*	Multidrug export protein	DEL	DEL	DEL
*16364_S1_01637*	Hypothetical protein	INS	SNP	SNP
*ypjD*	Inner membrane protein	SNP	SNP	SNP
*rpoS*	RNA polymerase sigma factor	DEL	DEL	DEL
*16364_S1_01843*	Hypothetical protein	SNP	SNP	SNP
*16364_S1_03573*	Hypothetical protein	DEL	DEL	NONE
*16364_S1_02660*	Hypothetical protein	INS	NONE	NONE
*16364_S1_03577*	16364_S1_03577	DEL	NONE	NONE
*barA*	Signal transduction histidine-protein kinase	SNP	NONE	NONE
*16364_S1_03563*	Hypothetical protein	NONE	DEL	DEL
*16364_S1_03578*	Hypothetical protein	NONE	DEL	NONE
**Oxidative stress**				
*PGdx_1*	Hybrid peroxiredoxin hyPrx5	DEL	NONE	NONE
*sasA_9*	Adaptive-response sensory-kinase	SNP	DEL	NONE
*sppA*	Protease 4	SNP	SNP	SNP
*dctB*	C4-dicarboxylate transport sensor protein	SNP	NONE	SNP
*34701_S35_01938*	Hypothetical protein	INS	NONE	INS
*caiA*	CaiA	DEL	DEL	DEL
*galS*	GalS	DEL	DEL	DEL
*barA*	Signal transduction histidine-protein kinase	SNP	SNP	SNP
*ompL_2*	Porin-like protein L	SNP	SNP	SNP
*luxR*	HTH-type transcriptional regulator	SNP	SNP	SNP
*34701_S35_03534*	Hypothetical protein	DEL	DEL	NONE
*34701_S35_02844*	Hypothetical protein	NONE	SNP	NONE
*ftsY_2*	Signal recognition particle receptor	NONE	NONE	DEL

^*a*^ None, non-mutated gene; SNP, single nucleotide polymorphism resulting in a non-synonymous codon; DEL, mutation causing a complete or partial gene deletion. The raw data from Snippy used for this analysis are found in Supplementary Data 2.

Many common SNPs were also observed across the three evolved isolates under oxidative stress (Table 4). Most of the affected genes encode global regulatory proteins such as LuxR and BarA, which might control functions related to adaptation to oxidative stress. The other common genes with SNPs are *sppA* (signal peptide peptidase), *caiA* (desulfurase involved in carnitine catabolism), *galS* (repressor of the galactose metabolic network), and *ompL2* (outer membrane porin). The role of these genes in the adaptation to oxidative stress is unclear, except in the case of *ompL*. In *Escherichia coli*, a role for OmpL in oxidative stress has been described (36), suggesting that porin may be required for the modulation of redox potential in the periplasmic space but a subsequent study could not confirm this function (37). Together, the combined analysis of SNPs and substrate utilization profiles suggests that while some genes may have a direct effect on the profiles observed, it is more likely that the mutations in regulatory proteins may account for most of the similarities and differences among the evolved variants.

### Virulence potential of the parental strains and evolved variants

The virulence potential of the parental strains (clinical and environmental) and their evolved variants were assessed using the *G. mellonella* larvae infection model. Initial experiments, inoculating 10^4^ cfu/mL bacteria resulted in 100% mortality of the infected larvae with all the strains. Further experiments at lower bacterial doses demonstrated that 10^2^–10^3^ cfu/mL of the environmental strains and their evolved variants caused 100% larvae mortality, while 10^3^ and 10^4^ cfu/mL were required for mortality when using the clinical strains and their evolved variants, suggesting that the environmental strains were more pathogenic in this model than the clinical strains. Reducing the bacterial inoculum to 10 and 10^2^ cfu/mL for environmental and clinical strains, respectively, allowed us to determine differences in virulence between the parental strains and their evolved variants. Generally, strains S1 (environmental) and E4 (environmental) showed high mortality rates (above 90%), which were not significantly different from their evolved variants (Fig. S8A and S9C). Strains E19 (environmental) and C6 (clinical) were significantly less pathogenic (28% mortality rates, respectively) than their evolved variants (between 33% and 98% mortality rates) (Fig. S8D and S9E). C17 (clinical), although highly pathogenic (73% mortality rate), was significantly less pathogenic than its evolved variants (88%–100% mortality rates) (Fig. S8F). C22 (clinical) was generally more virulent (95% mortality rate) than its evolved variants (25%–90% mortality rates) (Fig. S8G) and S35 (environmental) was also either more or less virulent (62% mortality rate) than its evolved variants (0% and 82% mortality rates, respectively) (Fig. S8B). The results indicate that environmental strains and their evolved variants were generally more pathogenic than clinical strains, as demonstrated by the lower CFU/mL needed to cause 100% mortality. These findings underscore the complexity of pathogenicity and the impact of environmental factors on virulence evolution.

## DISCUSSION

Long-term exposure of *V. cholerae* to multiple environmental stressors resulted in variants with various colony morphotypes which also exhibited increased hemolysis and biofilm formation, suggesting these properties may provide *V. cholerae* an advantage for stress survival and persistence, as found in other bacteria (38–40). The increased production of β**-**hemolytic activity by the evolved variants resembles previous observations with *V. cholerae*, which when passaged in the rabbit gut caused increased β-hemolysis and production of β-hemolysis in previously non-hemolytic strains of *Aeromonas* sp. and *V. cholerae* (41, 42). These observations could be explained by changes in gene expression induced by stress conditions like the rabbit’s gut microenvironment (41, 42). β-hemolytic strains exhibit enterotoxicity, which also aids in the pathogenesis of *V. cholerae* gastroenteritis (43).

The variations in the genome sizes of the evolved variants compared to the parental strains and the chromosomal rearrangements observed in this study suggest adaptive mechanisms in response to stress that may be important for bacterial survival or persistence both in the environment and eukaryotic hosts (44). The mutations in the EAL and HD-GYP domain-containing proteins, in the two distinct phylogenetic clusters containing a majority of the evolved variants underscores the importance of the ubiquitous second messenger, c-di-GMP in bacterial stress adaptation. The cytoplasmic levels of c-di-GMP are modulated by the relative activities of two classes of enzymes with opposite effects: GGDEF domain-containing diguanylate cyclases (DGC), which catalyzes c-di-GMP synthesis, and EAL or HD-GYP domain-containing phosphodiesterases (PDE), which hydrolyze c-di-GMP (27). Studies have shown that the balance of these opposing activities plays a critical role in bacterial adaptation to extracellular stimuli, involving changes in metabolism and control of cellular processes such as motility, biofilm formation, cell development, and virulence (45, 46). Mutations in these enzymes may also trigger the accumulation of c-di-GMP, due to the inability of either EAL or HD-GYP to break down c-di-GMP into pGpG and 2GMP, respectively. The intracellular accumulation of c-di-GMP induces increased expression of the *Vibrio* polysaccharide (*vps*) and mannose-sensitive haemagglutinin (*msh*) operons (47), which promotes biofilm formation and wrinkled colony morphotypes (48, 49), as observed especially in our cluster 3 strains.

Cluster 2 EAL-domain-containing proteins had their EAL domains linked to GGDEF domains. Proteins containing such dual domains have either, DGC, PDE, bifunctional DGC, and PDE activities or no enzymatic activity if either enzyme module has inactive motifs (50–53). This may account for the different biofilm-forming phenotypes (decreased, increased, and no difference) of the variants in cluster 2 compared to their parental strains. For example, *Azospirillum baldaniorum Sp245* GGDEF-EAL dual domain-containing protein CdgB exhibits both DGC and PDE activities and increases or decreases biofilm formation depending on the condition (54). BifA, a GGDEF-EAL dual domain-containing protein in *P. aeruginosa,* exhibits PDE activity and reduced biofilm formation (55). Furthermore, the N-terminal domains of the EAL and HD-GYP domain-containing proteins (transmembrane, PAS, PAC, and GAF; Fig. 3) are involved in modulating bacterial activities in response to external stimuli (56, 57). PAS domains help bacteria adapt to environmental changes by regulating gene expression and metabolic processes in response to external stimuli. PAC stabilizes the PAS domain structure and assists in the proper folding and function of PAS domain-containing proteins, while the GAF domains mediate responses to changes in cyclic nucleotide levels. Mutations in these domains likely play significant roles in the environmental stress survival of the *V. cholerae* strains. The *RS0084* gene encoding a GGDEF domain-containing protein is a recombination hotspot in the strains and evolved variants, further highlighting the importance of c-di-GMP in the long-term survival of the evolved variants.

Differences in substrate utilization of the parental strains and evolved variants may have provided longer survival advantages. Studies suggest a link between substrate utilization, stress survival, and virulence in microorganisms. For instance, variants of S1 displayed increased utilization of phosphorus and sulfur sources, while E19 variants showed selective nitrogen source utilization when passaged in high osmolarity and oxidative stress conditions. These metabolic changes may reflect stress-induced redirection of metabolic fluxes. While some direct correlations were found between substrate utilization and specific mutations, the presence of SNPs in regulatory proteins suggests that alterations in global regulons can underlie these differences. For example, mutations in *barA*, a global regulator involved in stationary phase adaptation, may explain S1’s ability to utilize multiple nitrogen sources (Fig. 4). Carbon metabolism is crucial to pathogen persistence and virulence (58, 59). Moreover, mutations in the quorum sensing master regulator LuxR, contribute to carbon metabolism and fitness in *Vibrio* sp. (60). The ability to utilize L-arabinose and pectin as sole carbon and nitrogen sources are associated with acid tolerance and biofilm formation, respectively, in *Salmonella enterica* serotypes (61, 62). The differential ability to metabolize turanose instead of sucrose has been associated with the high virulence phenotype of *Scedosporium aurantiacum*, an opportunistic pathogenic fungus (63). In uropathogenic *E. coli* strains, the ability to catabolize D-serine is also an indicator of increased pathogenic potential (59). Additionally, microbial stress survival benefits from the ability to metabolize glycine, α-ketobutyric acid, and D-mannose, as observed in our study. For example, α-ketobutyric acid metabolism in *E. coli* increases bacterial survival in manure-amended soils (64). Moreover, mannose utilization is important for pathogenic bacteria, as shown by the ability of pathogenic *E. coli* to utilize mannose and the observation that mutations in genes involved in mannose utilization cause reduced growth (65). Concerning the substrate utilization profiles, differences in the growth or no growth of a subset of evolved variants were demonstrated, but the results could not be directly correlated to genetic mutations in specific genes (SNPs and deletions). However, mutations were observed in several genes encoding global regulatory proteins, suggesting that the differences in substrate utilization may be due to the alteration of regulons. This is further supported by our observation of mutations in genes encoding proteins involved in the synthesis and degradation of c-di-GMP.

The dose curve experiments for *G. mellonella* infection indicated that both parental strains and evolved variants are highly pathogenic. A high mortality rate (84%) in *G. mellonella* was previously reported for clinical *V. cholerae* strains from O1 and non-O1 serotypes (66, 67). While our results agree with these previous studies, the increased virulence in *G. mellonella* of the environmental strains compared to the clinical strains suggests that the environmental strains are better suited to overcome the larvae’s innate immune defenses. However, the model is limited since it is not appropriate to assess the role of the cholera toxin, a major virulence factor for *V. cholerae,* and involves parenteral inoculation instead of intestinal colonization, the normal route of cholerae acquisition.

In summary, this study revealed that stress adaptation of clinical and environmental *V. cholerae* strains leads to variants with enhanced survival under challenging conditions. Mutations in genes regulating c-di-GMP levels likely drive many of these adaptations, influencing biofilm formation, motility, and colony morphology. Even though differences in substrate utilization between the parental strains and evolved variants appear to be minor, the metabolic changes observed could still play a role in stress survival and potential infection. These observations may explain why some *V. cholerae* strains such as the El-Tor biotype and variants have evolved over time, resulting in strains with better survival ability in the environment and in the human host. Our data also suggest that despite its limitations, *G. mellonella* may be a useful model to probe differences among environmental isolates in the context of survival in the environment and in association with non-human organisms.

## MATERIALS AND METHODS

### *Vibrio cholerae* strains

Six environmental (S1, S35, E4, E19, E30, and E32) *V. cholerae* strains used in this study (Table 5) were isolated from sampling sites in the Korle-Lagoon of the Greater Accra Region, Ghana, from 2016 to 2020 (68). As a case-control, we obtained three clinical strains (C22, C17, C6) from the Ghana National Public Health and Reference Laboratory. All strains were grown on lysogeny broth (LB) agar plates at 37°C for 18–24 h. To investigate colony morphotypes by visual inspection, strains were incubated at room temperature for an additional 24–48 h.

**TABLE 5:** *Vibrio cholerae* strains used in this study

Strain	NCBI accession	Year	Source	Serogroup	Biotype	Tax ID
S1	SAMN26804320	2019	Environmental(water)	Non O1/O139	N/A	666
S35	SAMN26804321	2020	Environmental(water)	Non O1/O139	N/A	666
E4	SAMN26804322	2016	Environmental(water)	Non O1/O139	N/A	666
E19	SAMN26804323	2016	Environmental(water)	Non O1/O139	N/A	666
E30	SAMN26804324	2016	Environmental(water)	Non O1/O139	N/A	666
E32	SAMN26804325	2016	Environmental(water)	Non O1/O139	N/A	666
C6	SAMN26804327	2010	Clinical(stool)	O1	El-Tor Ogawa	666
C17	SAMN26804329	2014	Clinical(stool)	O1	El-Tor Ogawa	666
C22	SAMN26804331	2011	Clinical(stool)	O1	El-Tor Ogawa	666

^*a*^ N/A, not applicable

### Protease and hemolytic activity

Protease activity was detected as described (69) using a *Bacillus* isolate as a positive control. Casein plates were prepared by mixing freshly autoclaved 3% skimmed milk (Sigma Aldrich, UK) in distilled water with an equal volume of warm 1% tryptone (Sigma Aldrich, UK), 0.5% yeast extract (Melford, UK), 1% NaCl (Sigma Aldrich, UK), and 3% BD Bacto agar. Ten microliter of exponential growing cell cultures (OD_600_ = 0.5) were spotted onto the casein plates and incubated at 37°C. Protease activity was assessed by measuring the diameters of zones of clearing around the colonies after 48 h incubation.

Hemolysin production was determined in blood agar plates (Mueller Hinton agar, Oxoid, UK +5% sheep blood) spotted with 10 µL aliquots of exponential growing cells after incubation at 37°C. Hemolytic activity was assessed by measuring the diameter of the hemolytic zone around the colonies at 48 h. The hemolytic *Staphylococcus aureus* ATCC 29133 was used as a control.

### Biofilm biomass

The method of O’Toole (70) was used with minor modifications. A 1:100 dilution of exponentially growing cells were prepared in a biofilm medium consisting of LB supplemented with 1 mM MgSO_4_ (Sigma-Aldrich, UK) and 0.4% arginine (Sigma Aldrich, UK). One hundred microliter aliquots were placed in 96 well microtiter plates and incubated at 37°C for 24 h. Plates were washed twice in water to detach unattached cells and stained with 125 µL 0.1% solution of crystal violet (Sigma Aldrich, UK) following incubation at room temperature for 10–15 min. Plates were rinsed with distilled water and blotted on a stack of paper towels to get rid of excess cells and dyes. Upon drying, 125 µL of 30% acetic acid (VWR chemicals, UK) in water was added to the wells to solubilize the crystal violet. Solubilized crystal violet was transferred to a new flat-bottomed microtiter plate and quantified at 600 nm absorbance. These assays were performed using a clinical *Pseudomonas aeruginosa* strain as a control.

### Experimental evolution under various stress conditions

Experimental evolution of the *V. cholerae* isolates was performed under various stress conditions (see below). Unless indicated otherwise all cultures were grown in low salt LB broth (Melford, UK; 1% Peptone, 0.5% Yeast extract, 0.5% NaCl) adjusted to a pH of 8.0. Bacterial survival was monitored for up to 200 days. Exponentially growing cells (OD_600_ = 0.5) were used to start the experimental evolution experiments by serial transfer (Fig. S1). For each stress condition or the LB negative control, the culture volume was 10 mL maintained in 50 mL Falcon tubes using a shaking incubator (120 rpm) at 37°C. Cultures were propagated every 96 h by transferring 0.1 mL of each culture into 9.9 mL of fresh medium. For every transfer an aliquot (1 mL) was frozen for continuation in case of contamination. Culture purity was checked at each transfer by subculturing onto LB agar. Iron stress conditions consisted of a solution of freshly prepared FeSO_4_ (HD chemicals, UK) in LB at a final concentration of 20 µM (iron-replete medium) and LB containing the iron chelator 2, 2′-dipyridyl (Sigma-Aldrich, UK) at a final concentration of 200 µM (iron-depleted medium). Oxidative stress was achieved in LB supplemented with hydrogen peroxide to a final concentration of 1 mM. Osmotic stress was achieved in 1% Tryptone (Sigma Aldrich), 0.5% Yeast extract (Melford, UK), and 3% NaCl (Sigma Aldrich, UK), pH 8.0. The acidic pH stress medium was LB adjusted to a pH of 6.0 with sulfuric acid (VWR chemicals) and LB pH 8.0 was used as a control. Viable *V. cholerae* strains were harvested on days 60, 140, and 200 for iron depleted and replete, days 60, 120, and 180 for osmotic and oxidative, and on days 60, 112, and 150 for pH stress conditions (Fig. S1). No viable strains were recovered after day 150 for pH stress.

### Whole genome sequencing

Genomic DNA was extracted using the QIAamp DNA Mini Kit, following the manufacturer’s instructions, and quantified using NanoDrop. Genome sequencing was done by MicrobesNG (Birmingham), and the genomic DNA library was prepared using the Nextera XT library prep kit (Illumina) according to the manufacturer’s protocol with the following modifications: 2 ng of DNA instead of one was used as input, and PCR elongation time was increased to 1 min from 30 s. DNA quantification and library preparation were carried out on a Hamilton Microlab STAR automated liquid handling system. Pooled libraries were quantified using the Kapa Biosystems Library Quantification Kit for Illumina on a Roche light cycler 96 qPCR machine. Libraries were sequenced on the Illumina HiSeq using a 250 bp paired-end protocol. Reads were adapter trimmed using Trimmomatic 0.30 with a sliding window quality cutoff of 15. *De novo* assembly was performed on samples using SPAdes version 3.7, and Contigs were annotated using Prokka 1.11 all via MicrobesNG (Birmingham). Sequence data for all strains used in this study were deposited in NCBI (accession numbers listed in Tables 1 and 5).

### Comparative genomics

SNPs, indels, and major insertions in the evolved variants against their corresponding parental strains were identified using Snippy (71). DNAdiff and MUMmer4 were used for whole genome sequence alignment to identify inversions, translocations, and breakpoints in the genomes. Genealogies unbiased by recombinations in nucleotide sequences (Gubbins) were used to analyze hotspots of recombination and to construct a phylogeny based on non-recombinant SNPs (25), using *V. cholerae* O1 biovar El Tor strain N16961 as the reference genome. The regions where clusters of SNPs deviate from the phylogenetic tree’s expected pattern suggest the SNPs occurred through recombination rather than point mutations. This is performed by looking for stretches of SNPs that appear in different strains but are inconsistent with the overall phylogeny inferred from the non-recombinant (core) regions. To detect this, Gubbins uses a statistical model to differentiate between mutations that occurred due to recombination versus those that occurred due to point mutations. It calculates the density of these recombination events along the genome to identify regions with a significantly higher density of recombination events. By examining the distribution of these recombination events across the genomes, the hotspots are identified as regions where recombination events are significantly more frequent than in other parts of the genome. This analysis returned an alignment of the non-recombinant SNPs (i.e., substitution mutations) and a maximum-likelihood phylogeny inferred from these SNPs, and each SNP was assigned either to a recombination block or a substitution event and to a branch on the phylogenetic tree. The output of the analysis was visualized with Phandango (72). The “unique SNPs” coordinates were detected, and the genes mutated in each phylogenetic cluster were identified by searching for the Unique SNPs prediction “Start and End coordinates” predicted by Gubbins, in the *V. cholerae* O1 biovar El Tor str. N16961 reference genome. Amino acid analysis to determine amino acid changes in the proteins encoded by mutated genes was done using Clustal Omega (73) and UGENE (74) for amino acid sequence alignment and SMART domain prediction server to determine the proteins domain architecture (75).

SNP analysis was also used to attempt a correlation with metabolic changes under each of the stress conditions used to evolve isolates in comparison with the evolution in LB only as a control. In this case, the trimmed paired-end reads were mapped to the reference genome of the respective ancestral strain for all samples using Snippy and Breseq (76). For strains sequenced at multiple time points, reads were also mapped to the genome of the immediately preceding evolutionary strain. After variant identification, a custom Python pipeline was applied to filter the results. This approach enabled us to compile a list of genes that accumulated SNPs during evolution in strains grown exclusively in LB medium without stress. The same workflow was applied to strains that evolved under specific stress conditions. Finally, the gene lists from both conditions were compared, retaining only the genes that presented mutations specifically under the stress condition.

### Metabolic phenotypic microarrays

Phenotypic microarray (PM) assays were performed using the Biolog system (Biolog, Inc., Hayward, CA), a tetrazolium-based growth assay (77) with plates PM1 and 2A (190 carbon sources), PM 3A (95 nitrogen sources), PM 4 (59 phosphorus and 35 sulfur sources), following the manufacturer’s instruction. Briefly, the *V. cholerae* strains and variants were revived from −80°C storage onto LB agar plates and grown overnight at 37°C. Single colonies were then sub-cultured onto LB agar plates and grown overnight at 37°C. Multiple colonies of each strain/variant were inoculated into IFOa GN/GP Base 1.2× medium plus sterile distilled water, to create a cell suspension of 42%T on a Biolog turbidimeter. The final inoculating fluid was prepared by mixing IFOa GN/GP Base 1.2× medium, Dye mix D, and sterile distilled water, with the 42%T cell suspension to yield a final cell density of 85%T. Each PM plate, PM 1 to PM 4, was inoculated in three biological replicates with 100 µL of the final cell suspension. Glycerol (5 mM) was added as a carbon source to the final inoculating fluid used for PM 3A and PM 4 plates. The PM plates were incubated at 37°C in an Omnilog incubator (Biolog) at 37°C for 48 h. Cell respiration was recorded every 15 min by a charge-coupled device camera and plotted as a kinetic curve depicting the reduction of the colorless tetrazolium redox dye to violet (formazan) by cell respiration. Negative controls were PM plate wells containing the inoculated growth medium without any substrate. These were subtracted from each reading for each plate. Data for each run was exported into csv files (PM data sets) using the Biolog Omnilog PM software in the parametric module. The PM data sets were analyzed using the R package software opm (78), whiihc was employed for curve parameter visualization and the automated generation of tabular reports (growth or no growth).

### *G. mellonella* larvae infection assay

Four environmental (S1, S35, E4, E19) and three clinical (C6, C17, C22) strains were selected for the *G. mellonella* infection assay based on the long-term viability (180 or 200 days depending on the stress condition) of their evolved variants (*n* = 30) in multiple stress conditions (Table 1). *G. mellonella* larvae (Waxworms Limited, Sheffield) of approximate weight of 250–350 mg were selected. *V. cholerae* strains and variants were retrieved from −80°C storage and grown overnight on LB agar at 37°C. An isolated colony was inoculated into 5 mL of LB broth and incubated at 37°C with shaking overnight. An aliquot of the overnight culture 150 µL was transferred to 15 mL of LB broth and grown to an OD_600_ = 0.5. Bacteria cells were adjusted to 10^2^ cfu/mL (clinical strains and variants) and 10 cfu/mL (environmental strains and variants) in PBS, and 10 µL of these concentrations were used for *Galleria* infection. Larvae surfaces were disinfected using 70% (vol/vol) ethanol and bacterial injections were done into the right proleg using a Hamilton syringe with a 30-gauge needle. Infected larvae (10 per group) were incubated at 37°C in 90 mm Petri dishes layered with 90 mm Whatman filter paper and survival was scored at 24, 48, and 72 h post infection. Death of larvae was confirmed by melanization and lack of movement even when gently probed with a Pasteur pipette. Controls were injected with 10 µL of PBS. Survival percentages (%) from three independent experiments were combined and comparisons were made between parental strains (day 0) and corresponding evolved variants (harvested on days 60 through 200, depending on the stress condition).

### Statistical analyses

All phenotypic assays were performed using at least three biological replicas with three technical repeats. For experimental evolution, GraphPad Prism software version 9 was used for statistical analysis to compare the means between parental strains (day 0) and evolved variants (strains harvested at days 60 through 200 depending on the stress condition) by unpaired two-tailed Student’s t tests, assuming both populations have same SD in parametric mode. Survival curves of *G. mellonella* larvae exposed to parental and evolved variants were generated using the Kaplan-Meier method and the Log-rank (Mantel-Cox) test used for statistical analysis. The 95% confidence interval (*P* value) from the Log-rank (Mantel-Cox) test, as upper and lower bands for each curve were used to indicate error bars. Survival curves resulting in *P* values of <0.05 relative to the unevolved or parental strains were considered significantly different.

## Data Availability

DNA sequences described in this work have been deposited in NCBI and the accession numbers are indicated in Tables 1 and 2. Any other additional data will become available from the authors upon request.

## SUPPLEMENTAL MATERIAL

Supplemental Data 1 spectrum.01211-24-s0001.xlsx SNP analysis for phylogeny.

Supplemental Data 2 spectrum.01211-24-s0002.xlsx SNP analysis to compare among stress conditions.

Supplemental figures spectrum.01211-24-s0003.pdf Fig. S1 to S8.

ASM does not own the copyrights to Supplemental Material that may be linked to, or accessed through, an article. The authors have granted ASM a non-exclusive, world-wide license to publish the Supplemental Material files. Please contact the corresponding author directly for reuse.
