# Supplementary material for: Stress adaptation under in vitro evolution influences survival and metabolic phenotypes of clinical and environmental strains of Vibrio cholerae El-Tor
Source: Microbiol Spectr. 2025 Feb 11;13(3):e01211-24. doi: 10.1128/spectrum.01211-24 (PMC11878068; doi:10.1128/spectrum.01211-24)
Supplement: Supplemental figures — Fig. S1 to S8. [file spectrum.01211-24-s0003.pdf]

SUPPLEMENTARY FIGURES

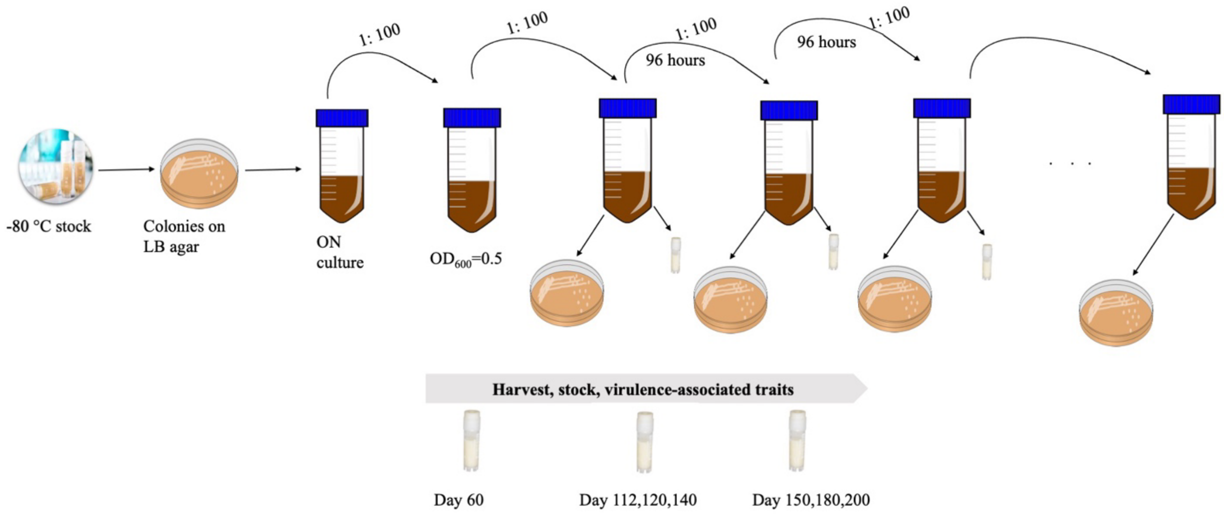

Figure S1: Schematic representation of the experimental evolution setup and harvest time points employed in this study. Serial transfer was done every 96 hours for up to 200 days. Viable *V. cholerae* strains were harvested on days 60, 140 and 200 for iron depleted and replete conditions and on days 60, 120 and 180 for osmotic and oxidative and on days 60, 112 and 150 for pH stress conditions. The harvested colonies were either stored as stock in – 80 °C or used for analysis of virulence-associated traits.

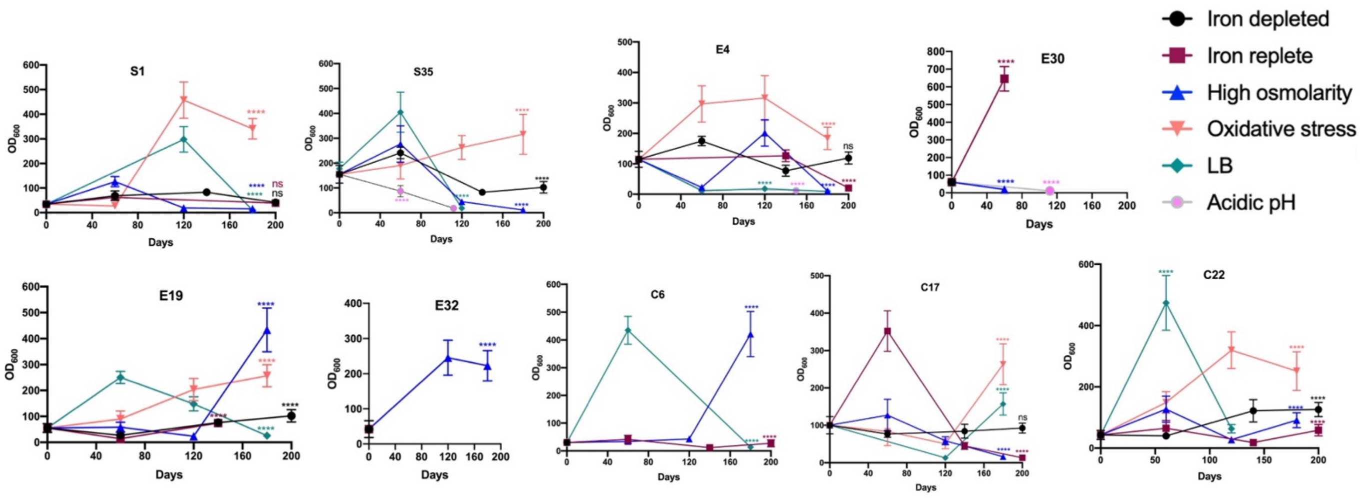

Figure S2: Biofilm formation of the *Vibrio cholerae* parental strains (Day 0), S1, S35, E4, E30, E19, E32, C6, C17, C22 and their respective evolved variants (strains harvested/viable at the different time points) (Days 60 through 200). (****p<0.0001). GraphPad Prism software version 9 was used for statistical analysis to compare the means between parental strains (Day 0) and evolved variants by unpaired two-tailed student's t tests, assuming both populations have same SD in parametric mode.

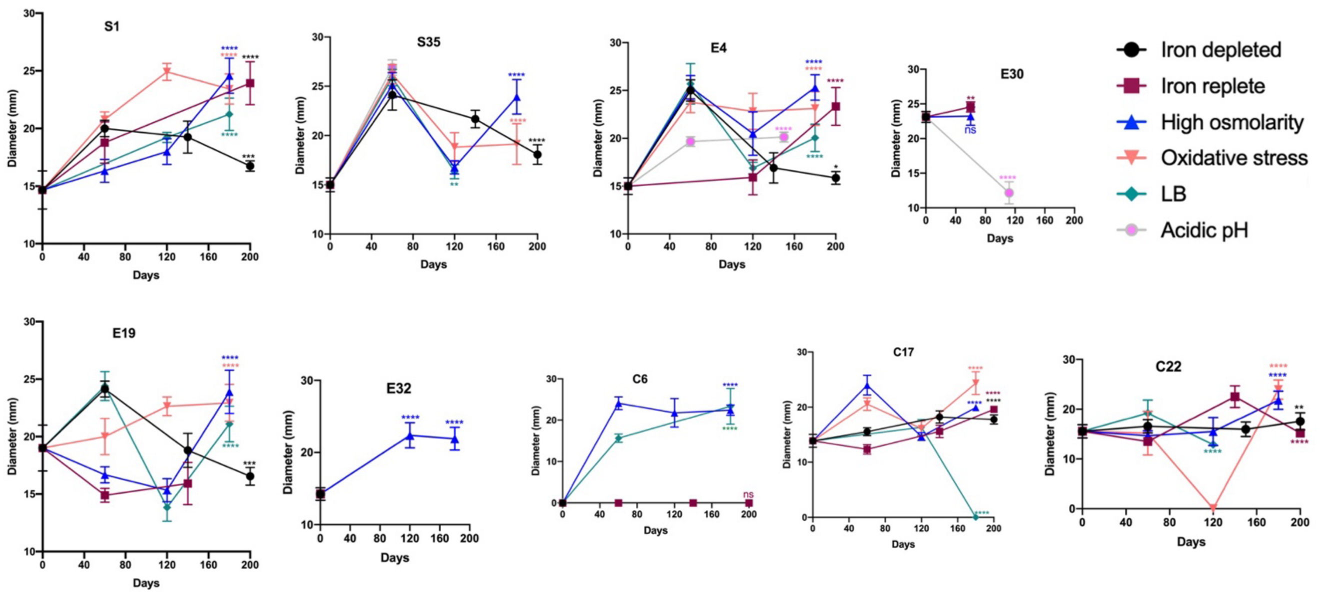

Figure S3: Haemolytic activity of the *Vibrio cholerae* parental strains (Day 0), S1, S35, E4, E30, E19, E32, C6, C17, C22 and their respective evolved variants (strains harvested/viable at the different time points). (Days 60 through 200). (****p<0.0001). GraphPad Prism software version 9 was used for statistical analysis to compare the means between parental strains (Day 0) and evolved variants by unpaired two-tailed student's t tests, assuming both populations have same SD in parametric mode.

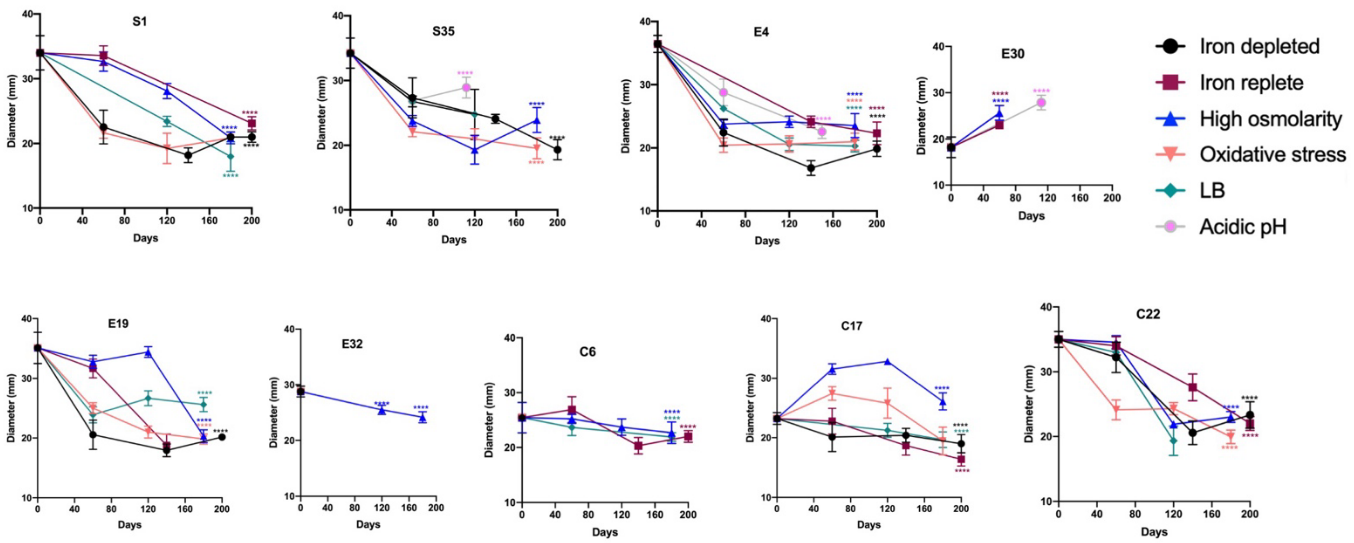

Figure S4: Protease activity of the *Vibrio cholerae* parental strains (Day 0), S1, S35, E4, E30, E19, E32, C6, C17, C22 and their respective evolved variants (strains harvested/viable at the different time points). (Days 60 through 200). (****p<0.0001). GraphPad Prism software version 9 was used for statistical analysis to compare the means between parental strains (Day 0) and evolved variants by unpaired two-tailed student's t tests, assuming both populations have same SD in parametric mode.

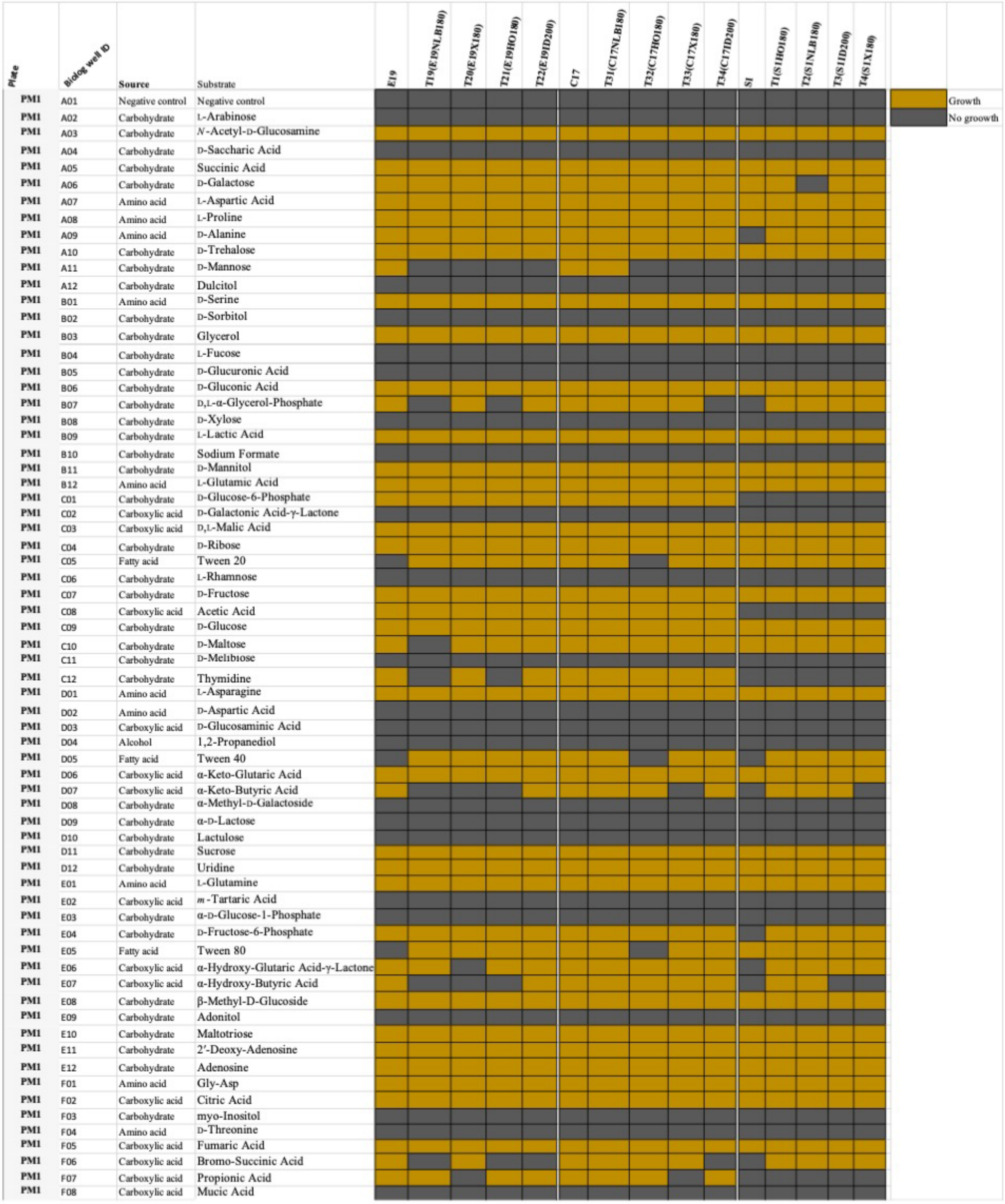

Figure S5A: Carbon substrates utilization of the parental *V. cholerae* strains and their evolved variants. The evolved variants were labelled combining the strain designation, stress conditions (X, oxidative stress; ID, iron depleted; HO, osmotic stress; IR-iron replete) and harvest time in days. The T designation represents the strain number assigned to each variant to facilitate the downstream analyses.

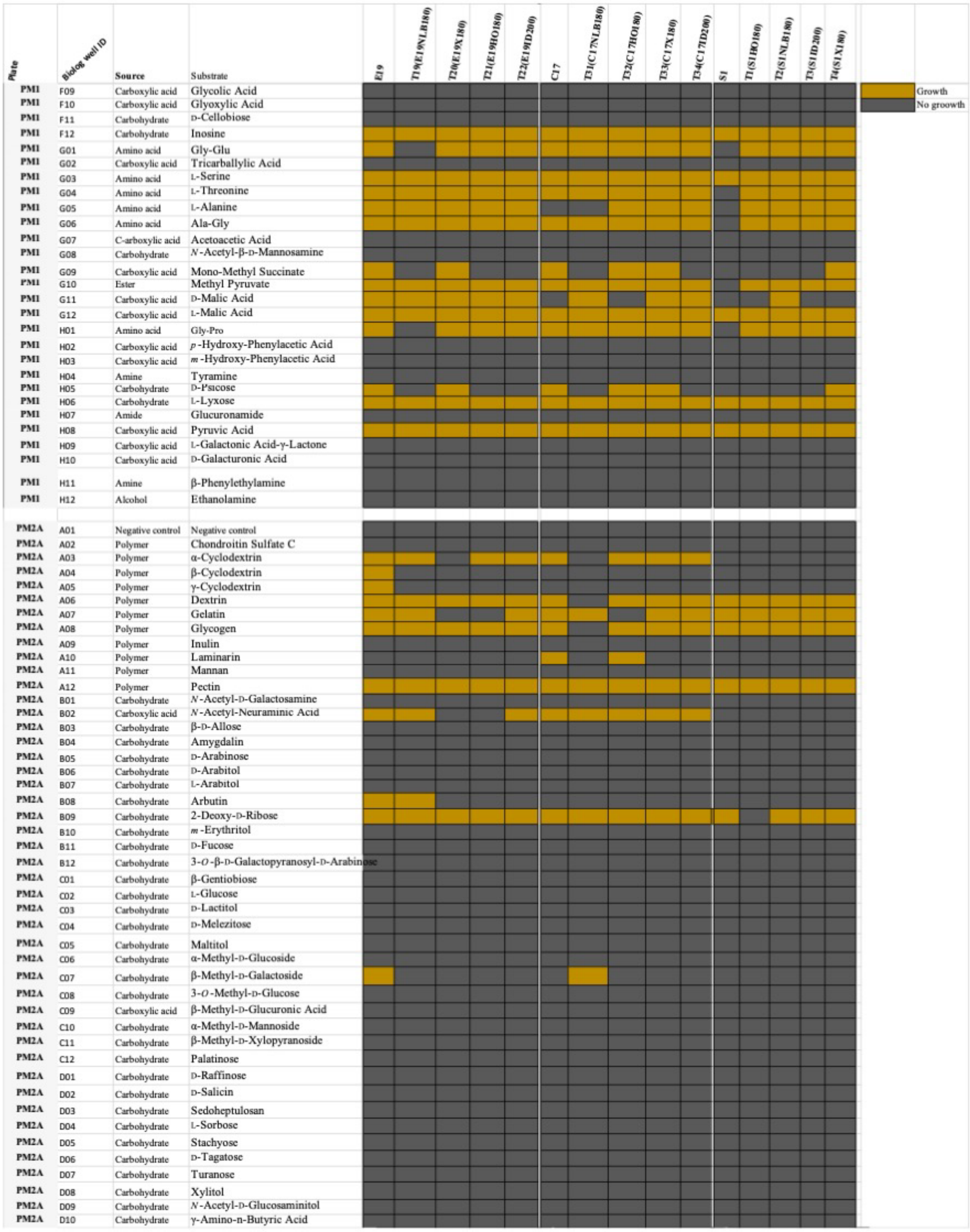

Figure S5B: Carbon substrates utilization of the unevolved *V. cholerae* strains and their evolved variants. The evolved variants were labelled combining the strain designation, stress conditions (X, oxidative stress; ID, iron depleted; HO, osmotic stress; IR-iron replete) and harvest time in days. The T designation represents the strain number assigned to each variant to facilitate the downstream analyses.

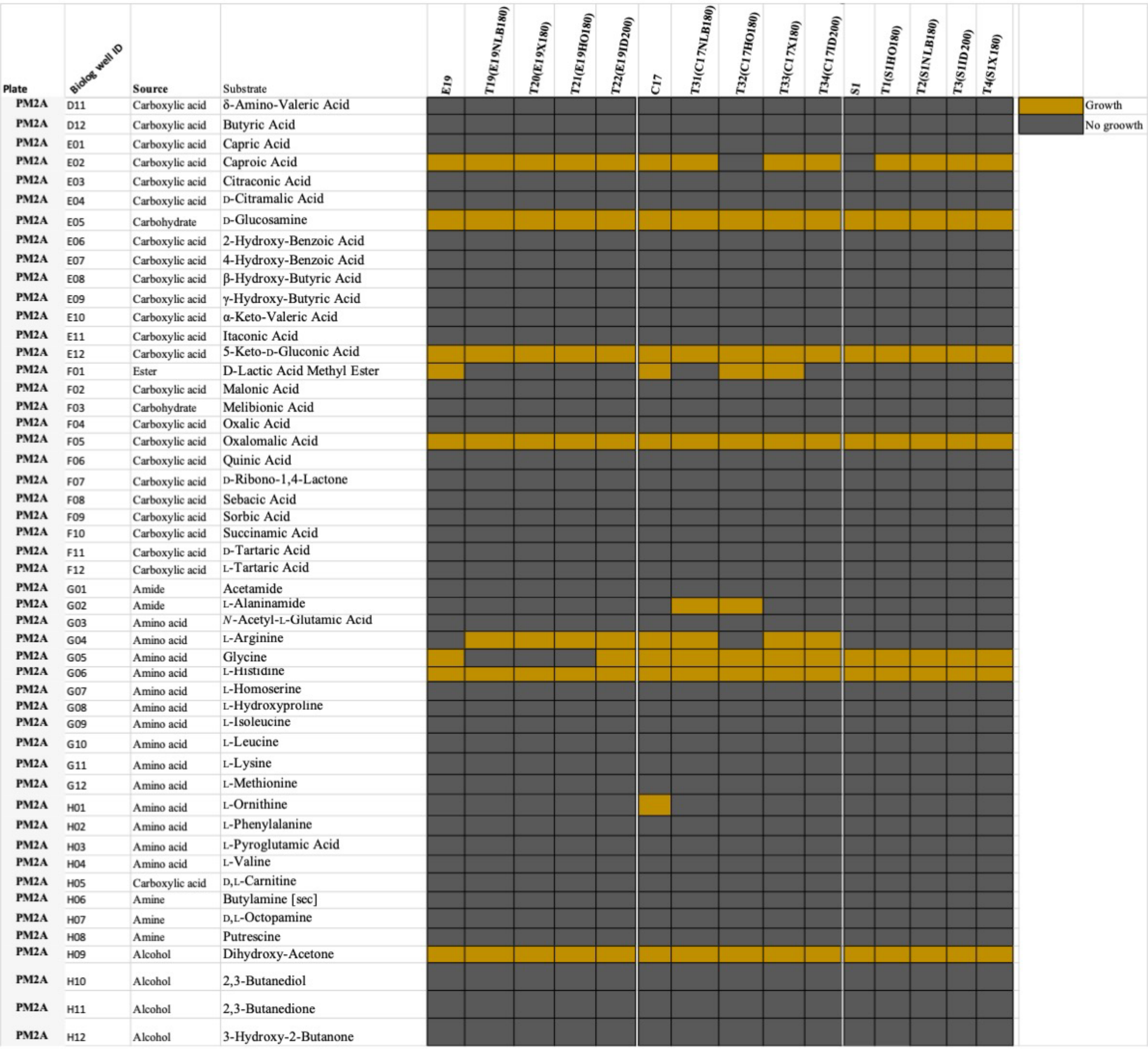

Figure S5C: Carbon substrates utilization of the unevolved *V. cholerae* strains and their evolved variants. The evolved variants were labelled combining the strain designation, stress conditions (X, oxidative stress; ID, iron depleted; HO, osmotic stress; IR-iron replete) and harvest time in days. The T designation represents the strain number assigned to each variant to facilitate the downstream analyses.

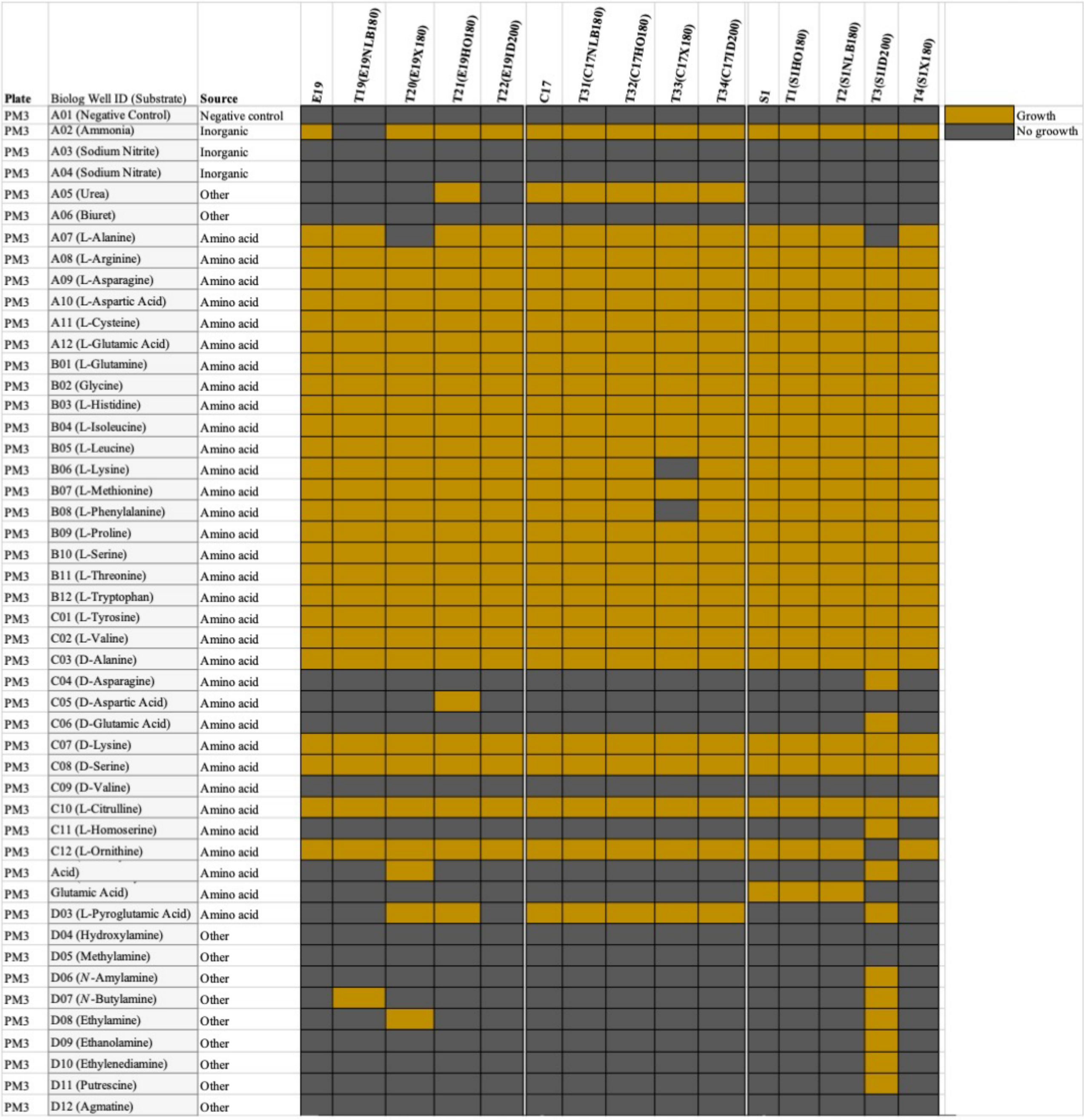

Figure S6A: Nitrogen substrates utilization of the unevolved *V. cholerae* strains and their evolved variants. The evolved variants were labelled combining the strain designation, stress conditions (X, oxidative stress; ID, iron depleted; HO, osmotic stress; IR-iron replete) and harvest time in days. The T designation represents the strain number assigned to each variant to facilitate the downstream analyses.

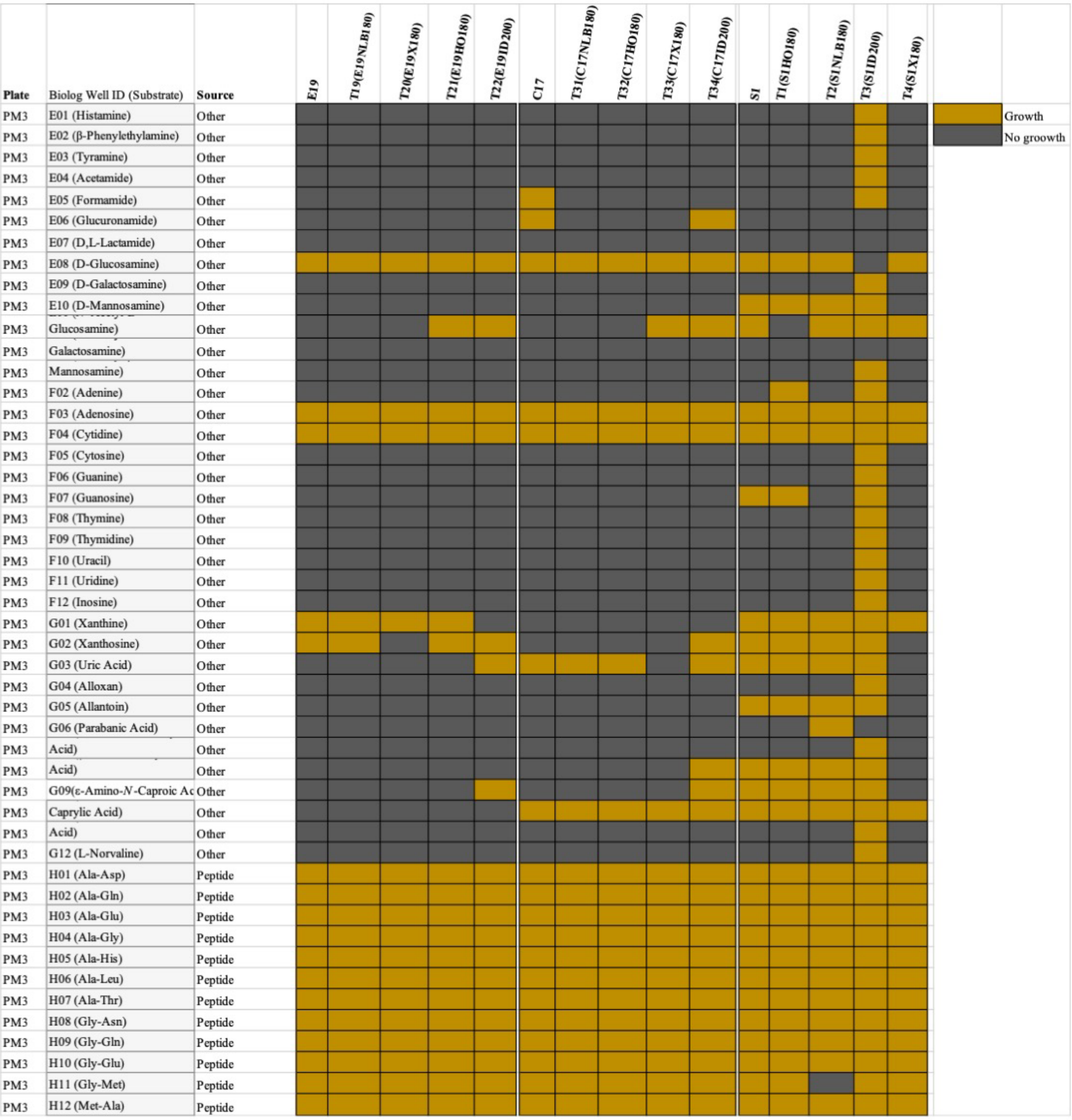

Figure S6B: Nitrogen substrates utilization of the unevolved *V. cholerae* strains and their evolved variants. The evolved variants were labelled using the strains, stress conditions and harvest time points. The evolved variants were labelled combining the strain designation, stress conditions (X, oxidative stress; ID, iron depleted; HO, osmotic stress; IR-iron replete) and harvest time in days. The T designation represents the strain number assigned to each variant to facilitate the downstream analyses.

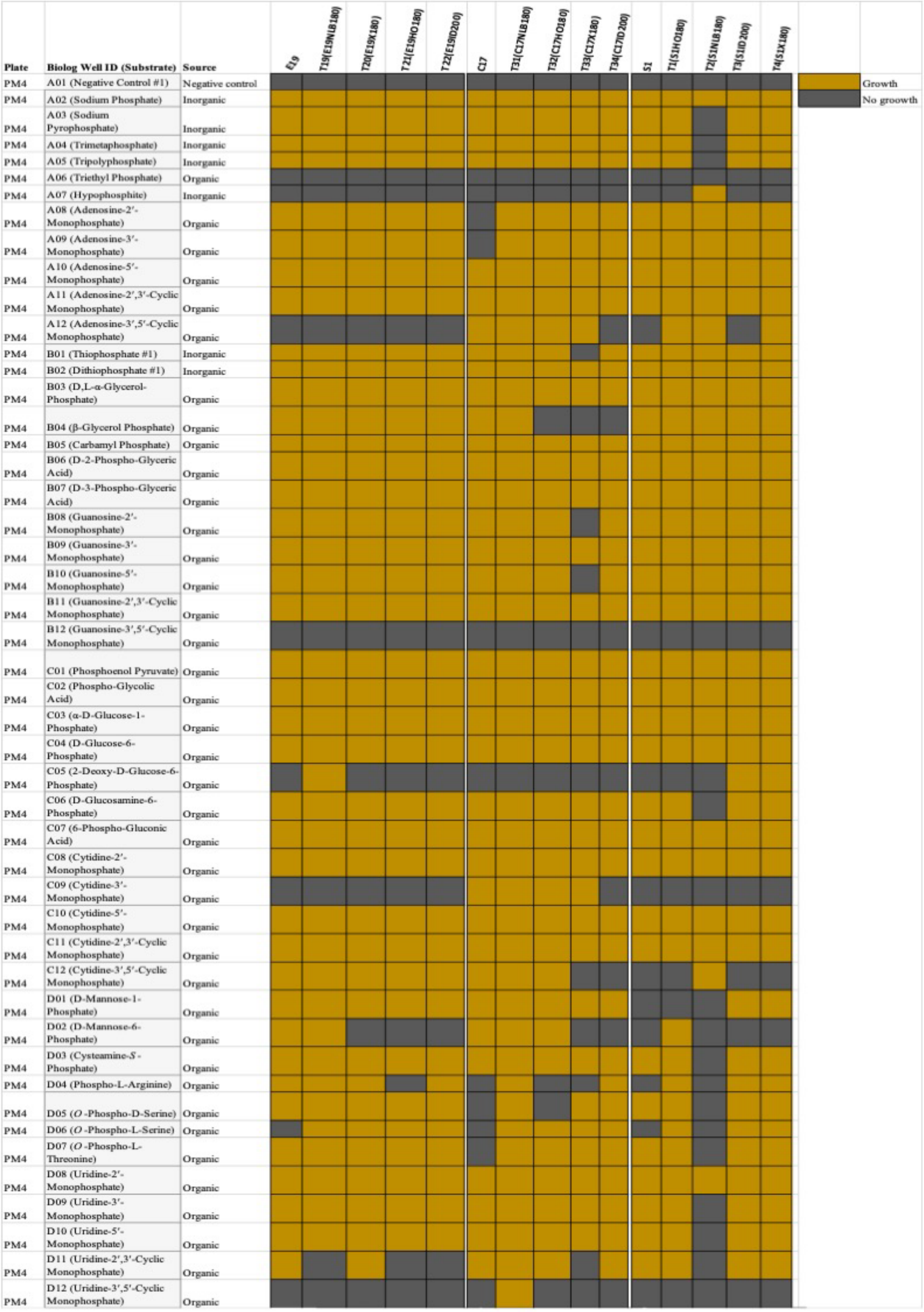

Figure S7A: Phosphorous and Sulphur substrates utilization of the unevolved *V. cholerae* strains and their evolved variants. The evolved variants were labelled combining the strain designation, stress conditions (X, oxidative stress; ID, iron depleted; HO, osmotic stress; IR-iron replete) and harvest time in days. The T designation represents the strain number assigned to each variant to facilitate the downstream analyses.

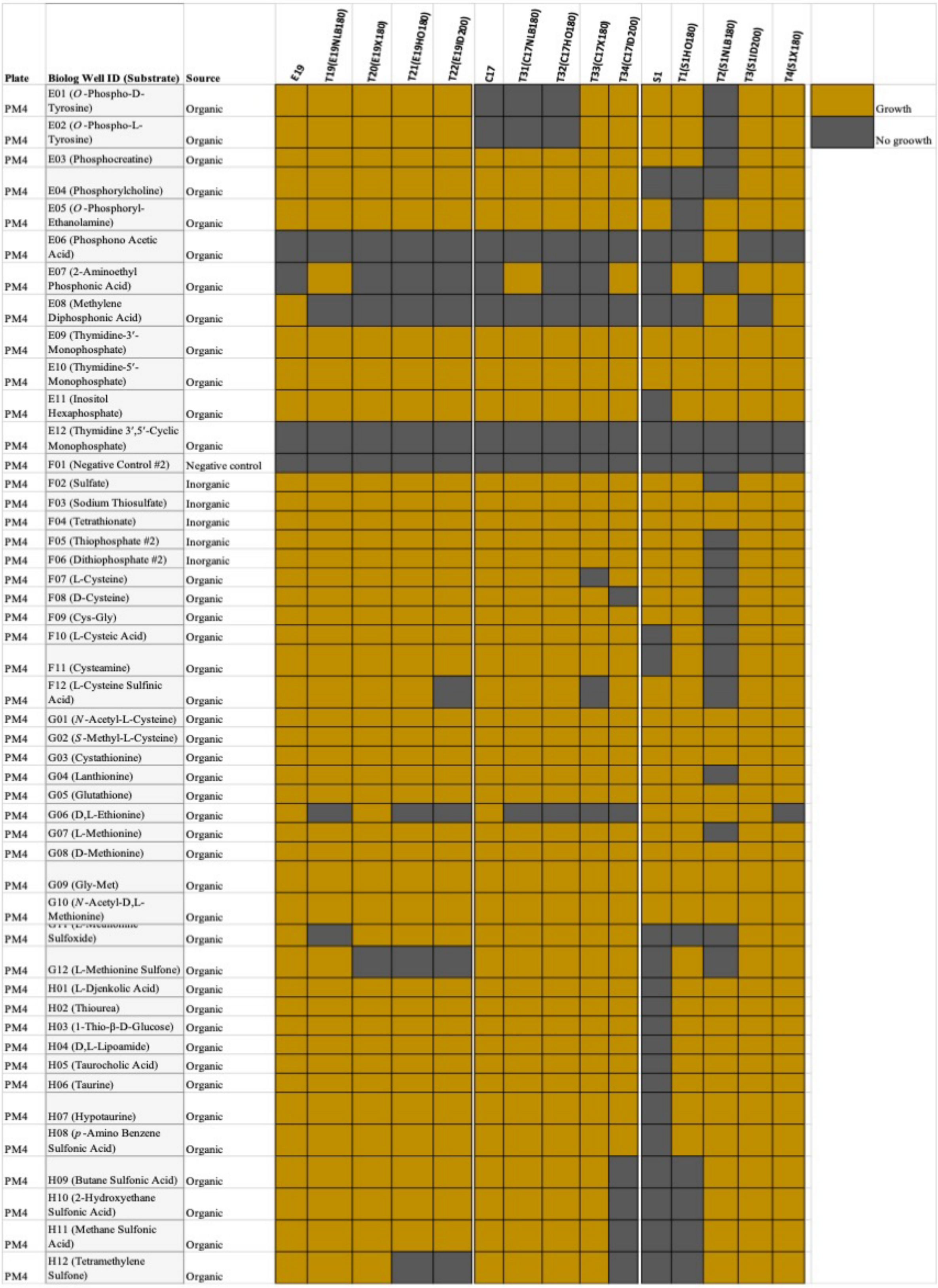

Figure S7B: Phosphorous and Sulphur substrates utilization of the unevolved *V. cholerae* strains and their evolved variants. The evolved variants were labelled combining the strain designation, stress conditions (X, oxidative stress; ID, iron depleted; HO, osmotic stress; IR-iron replete) and harvest time in days. The T designation represents the strain number assigned to each variant to facilitate the downstream analyses.

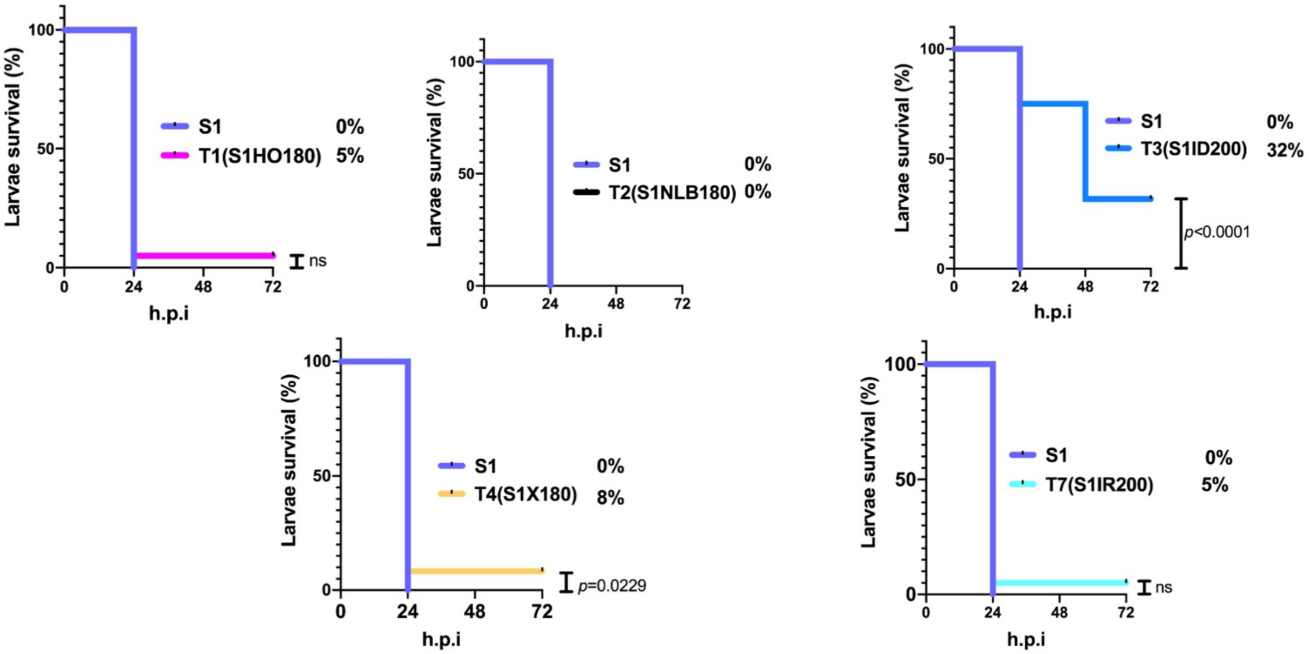

Figure S8A: Pairwise comparison of the survival rate of *G. mellonella* larvae infected with parental S1 (environmental) and evolved variants (T1, T2, T3, T4 & T7). S1 caused a high mortality rate that was not significantly different form (**A**). T1 (95%). (**B**). T2 (100%). S1 caused a significantly higher mortality rate (100%) than (**C**). T3(68%). (**D**). T4 (92%) (**E**). S1 and T7 caused very high mortality rates (100% and 95%) with no significant difference between them. (p<0.05). The evolved variants were labelled combining the strain designation, stress conditions (X, oxidative stress; ID, iron depleted; HO, osmotic stress; IR-iron replete) and harvest time in days. The T designation represents the strain number assigned to each variant to facilitate analyses.

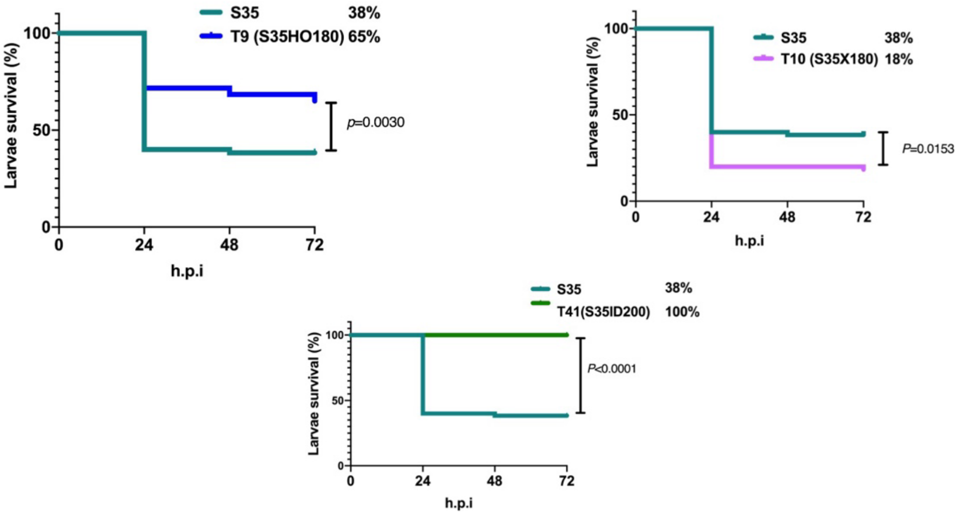

Figure S8B: Pairwise comparison of the survival rate of *G. mellonella* larvae infected with parental S35 (environmental) and evolved variants (T9, T10 & T41). **A**). S35 caused a significantly higher mortality rate (62%) than T9 (35%). **(B)** S35 caused a significantly lower mortality rate (62%) than T10 (82%) (**C**) S35 caused a significantly lower mortality rate than T41 (100%) (p<0.05). The evolved variants were labelled combining the strain designation, stress conditions (X, oxidative stress; ID, iron depleted; HO, osmotic stress; IR-iron replete) and harvest time in days. The T designation represents the strain number assigned to each variant to facilitate analyses.

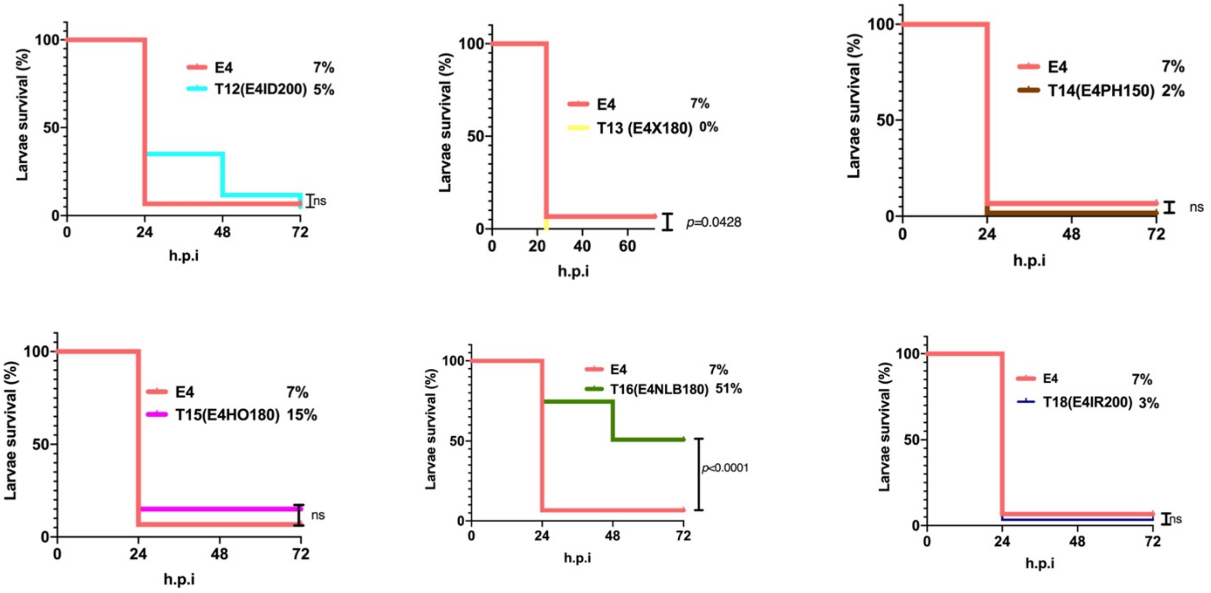

Figure S8C: Pairwise comparison of the survival rate of *G. mellonella* larvae infected with parental S35 (environmental) and evolved variants. (T12, T13, T14, T15, T16 & T18). (**A**) E4 and T12 caused high mortality rates (93% and 95%) which were not significantly different. (**B)**. E4 caused a significantly lower mortality rate (93%) than T13(100%). (**C**). E4 and T14 caused mortality rates (93% and 98%) that were not significantly different. (**D**). E4 and T15 caused high mortality rates (93% and 85%) that were not significantly different. (**E**). E4 caused a significantly higher mortality rate (97%) than T16(49%). (**F**). E4 and T18 caused high mortality rates (93% and 97%) that were not significantly different. (p<0.05). The evolved variants were labelled combining the strain designation, stress conditions (X, oxidative stress; ID, iron depleted; HO, osmotic stress; IR-iron replete) and harvest time in days. The T designation represents the strain number assigned to each variant to facilitate analyses.

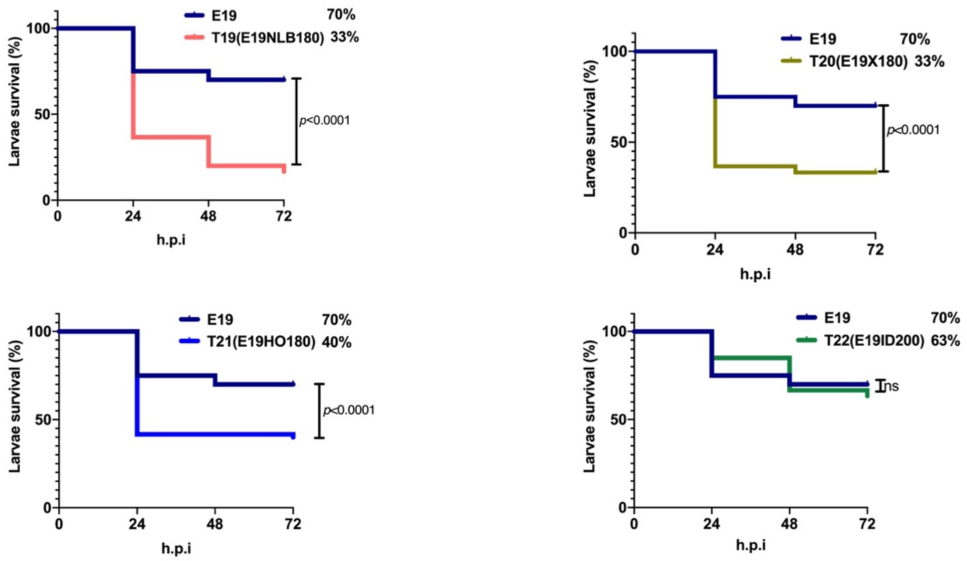

Figure S8D: Pairwise comparison of the survival rate of *G. mellonella* larvae infected with parental E19 (environmental) and evolved variants (T19, T20, T21, & T22). E19 caused a significantly lower mortality rate (30%) than (**A**) T19 (67%). (**B**). T20 (67%). (**C**). T21 (60%). (**D**). E19 and T22 caused mortality rates (30% and 37%) that were not significantly different. (p<0.05). The evolved variants were labelled combining the strain designation, stress conditions (X, oxidative stress; ID, iron depleted; HO, osmotic stress; IR-iron replete) and harvest time in days. The T designation represents the strain number assigned to each variant to facilitate analyses.

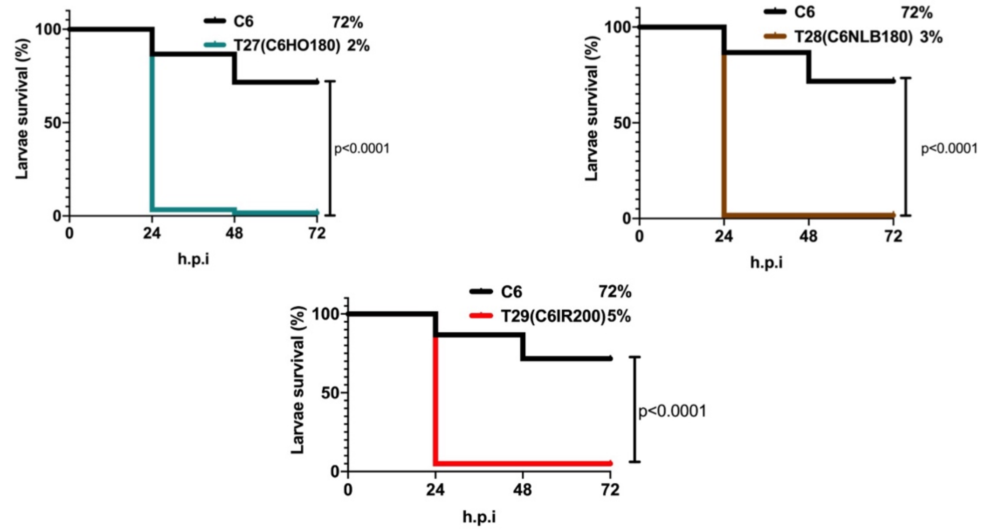

Figure S8E: Pairwise comparison of the survival rate of *G. mellonella* larvae infected with parental C6 (clinical) and evolved variants. (T27, T28 & T29). C6 caused a significantly lower mortality rate (28%) than all its evolved variants (**A)** T27 (98%). (**B**) T28 (97%) and (**C**) T29 (95%) (p<0.05). The evolved variants were labelled using the strains, stress conditions and harvest time points. The evolved variants were labelled combining the strain designation, stress conditions (X, oxidative stress; ID, iron depleted; HO, osmotic stress; IR-iron replete) and harvest time in days. The T designation represents the strain number assigned to each variant to facilitate analyses.

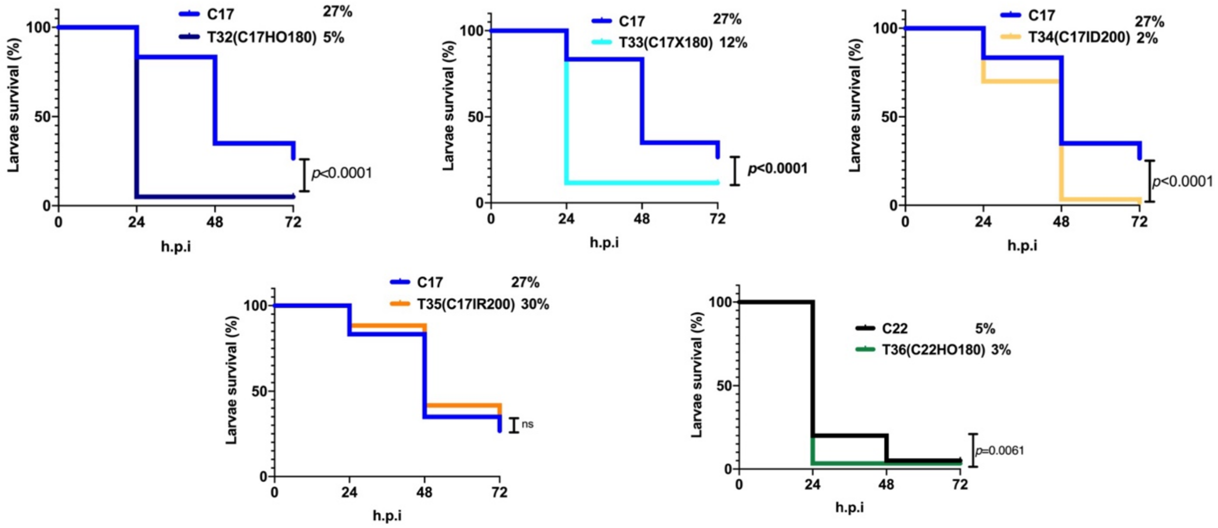

Figure S8F: Pairwise comparison of the survival rate of *G. mellonella* larvae infected with parental C17 (clinical) and evolved variants (T31, T32, T33, T34 & T35). C17 caused a significantly lower (73%) mortality rate than (**A**). T31 (100%). (**B**). T32 (95%). (**C**). T33 (88%). and (**D).** T34 (98%). (**E**). C17 and T35 caused mortality rates that were not significantly different (73% and 70%). (p<0.05). The evolved variants were labelled combining the strain designation, stress conditions (X, oxidative stress; ID, iron depleted; HO, osmotic stress; IR-iron replete) and harvest time in days. The T designation represents the strain number assigned to each variant to facilitate analyses.

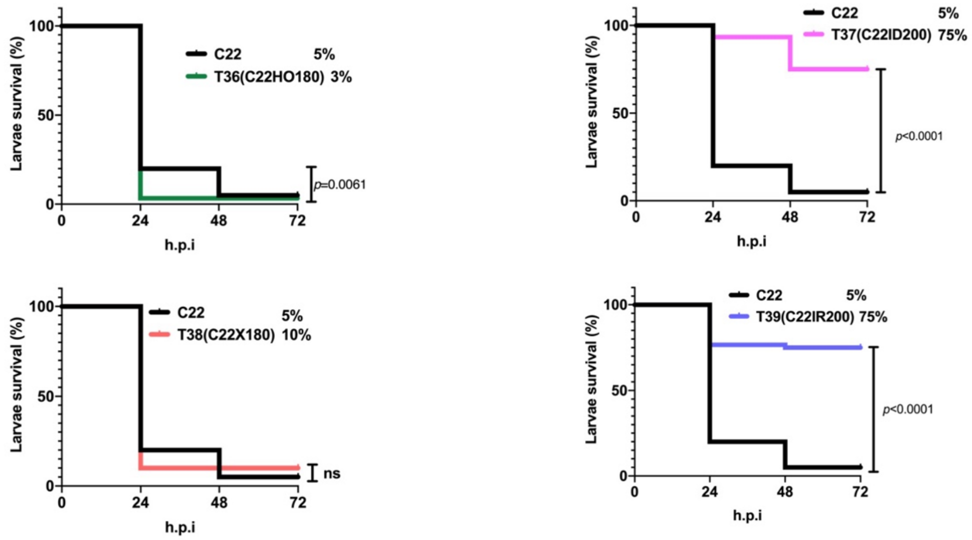

Figure S8G: Pairwise comparison of the survival rate of *G. mellonella* larvae infected with parental C6 (clinical) and evolved variants (T36, T37, T38, & T39). (**A)** C22 caused a significantly lower mortality (95%) rate than T36 (97%). (**B**). C22 caused a significantly higher mortality rate (95%) than T37(25%). (**C**). C22 and T38 caused mortality rates that were not significantly different (95% and 90%). (**D**). C22 caused a significantly higher mortality rate than T39 (25%). ($p<0.05$). The evolved variants were labelled combining the strain designation, stress conditions (X, oxidative stress; ID, iron depleted; HO, osmotic stress; IR-iron replete) and harvest time in days. The T designation represents the strain number assigned to each variant to facilitate analyses.
